# Approximate Monotone Local Search for Weighted Problems

**DOI:** 10.1007/s00453-026-01402-7

**Published:** 2026-08-18

**Authors:** Barış Can Esmer, Ariel Kulik, Dániel Marx, Daniel Neuen, Roohani Sharma

**Affiliations:** 1 CISPA Helmholtz Center for Information Security, Saarbrücken, Germany; 2 TU Dresden, Dresden, Germany; 3 DIMAG, Institute for Basic Science, Dajeon, South Korea; 4 Saarbrücken Graduate School of Computer Science, Saarbrücken, Germany; 5 Department of Industrial Engineering and Management, Ben-Gurion University of the Negev, Be’er Sheva, Israel

**Keywords:** Parameterized approximation, Monotone local search, Subset minimization problems, Approximation algorithm

## Abstract

In a recent work, Esmer et al. describe a simple method – approximate monotone local search – to obtain exponential approximation algorithms from existing parameterized exact algorithms, polynomial-time approximation algorithms and, more generally, parameterized approximation algorithms. In this work, we generalize those results to the weighted setting. More formally, we consider monotone subset minimization problems over a weighted universe of size *n* (e.g., Vertex Cover, *d*-Hitting Set and Feedback Vertex Set). We consider a model where the algorithm is only given access to a subroutine that finds a solution of weight at most $\alpha \cdot W$ (and of arbitrary cardinality) in time $c^k \cdot n^{\mathcal {O}(1)}$ where *W* is the minimum weight of a solution of cardinality at most *k*. In the unweighted setting, Esmer et al. determine the smallest value *d* for which a β-approximation algorithm running in time $d^{n + o(n)}$ can be obtained in this model. We show that the same dependencies also hold in a weighted setting in this model: we obtain a β-approximation algorithm running in time $d^{n + o(n)}$, for the same *d* as in the unweighted setting. Similarly, we also extend a β-approximate brute-force search (in a model which only provides access to a membership oracle) to the weighted setting. Using existing approximation algorithms and exact parameterized algorithms for weighted problems, we obtain the first exponential-time β-approximation algorithms that are better than brute force for a variety of problems including Weighted Vertex Cover, Weighted *d*-Hitting Set, Weighted Feedback Vertex Set and Weighted Multicut.

## Introduction

In this work, we are interested in *subset problems*, where the goal is to find a subset of a given *n*-sized universe *U* that satisfies some property Π (e.g., Vertex Cover, Hitting Set, Feedback Vertex Set, Multicut). Such problems can trivially be solved in time $\mathcal {O}^*(2^n)$^1^ (whenever Π is recognizable in polynomial time), and in the past decades there has been great interest in designing algorithms that beat this exhaustive search and run in time $\mathcal {O}^*\left( d^n\right)$ for as small 1<d<2 as possible (see, e.g., [1]). On the other hand, many of the considered problems admit polynomial-time α-approximation algorithms for some constant α>1 (e.g., Vertex Cover admits a polynomial-time 2-approximation [2]). To bridge the gap between exact exponential-time algorithms and polynomial-time α-approximation algorithms for some possibly large constant α, there has been recent interest in *exponential-time approximation algorithms* [3–10] to obtain approximation ratios that are better than what is considered possible in polynomial time.

In a recent work, Esmer et al. [3] describe a simple method – approximate monotone local search – to obtain exponential-time approximation algorithms for certain subset problems from existing parameterized exact algorithms, polynomial-time approximation algorithms and, more generally, parameterized approximation algorithms. More precisely, the focus in [3] lies on *subset minimization problems* where, given a universe *U* with *n* elements, we are aiming to find a set S⊆U of minimum cardinality satisfying some property Π. To allow for approximation algorithms, we restrict to *monotone* problems, i.e., the family ${\mathcal {F}}$ of solution sets is closed under taking supersets. In this setting, a β*-approximation algorithm* is asked to return a solution set $S \in {\mathcal {F}}$ such that $|S| \le \beta \cdot |\texttt {OPT}|$ where $\texttt {OPT}$ denotes a solution of minimum size. Given oracle access to a parameterized α-approximation algorithm running in time $\mathcal {O}^*(c^k)$ (where the parameter *k* denotes the size of the desired optimal solution), Esmer et al. [3] determine a value $d = \text {\texttt {amls}}(\alpha ,c,\beta )$ and obtain a β-approximation algorithm running in time $d^{n + o(n)}$. Additionally, in a suitable oracle model (see Sect. 2 for details), this running time is optimal (up to sub-exponential factors). Using existing parameterized approximation algorithms, which in particular include polynomial-time approximation and exact parameterized algorithms, this leads to the fastest exponential-time approximation algorithms for a variety of problems including Vertex Cover, *d*-Hitting Set, Feedback Vertex Set and Odd Cycle Transversal.

In this work, we are interested in *weighted* monotone subset minimization problems. Here, the universe *U* is additionally equipped with a weight function $\textbf{w}:U \rightarrow \mathbb {N}$ and we are asking for a solution set $S \in {\mathcal {F}}$ of minimum weight $\textbf{w}(S) :=\sum _{u \in S} \textbf{w}(u)$. Accordingly, in a β-approximation algorithm, we are seeking a solution set $S \in {\mathcal {F}}$ such that $\textbf{w}(S) \le \beta \cdot \textbf{w}(\texttt {OPT})$ where $\texttt {OPT}$ denotes a solution of minimum weight. Looking at [3], it can be observed that the obtained algorithms only extend to the weighted setting for the special case $\alpha = \beta = 1$. Indeed, in this particular case Fomin, Gaspers, Lokshtanov and Saurabh [11] already show in an earlier work that an exact parameterized algorithm running in time $\mathcal {O}^*(c^k)$ can be turned into an exact exponential-time algorithm running time $\mathcal {O}^*((2-\frac{1}{c})^n)$. As already pointed out in [11], the result also holds in a weighted setting and implies exact exponential-time algorithms for, e.g., *d*-Hitting Set and Feedback Vertex Set. On the other hand, for β>1, the algorithm presented in [3] does not work in a weighted setting. In a nutshell, the main idea in [3] is to randomly sample a set of vertices *X* and to bound the probability that it contains a sufficiently large portion of an optimum solution $\texttt {OPT}$. However, in a weighted setting, such an approach is destined to fail since even adding a single element of large weight to an optimum solution may lead to an unbounded approximation factor.

The main contribution of this work is to adapt the tools from [3] to the weighted setting. Given a parameterized α-approximation algorithm running in time $\mathcal {O}^*(c^k)$ for a weighted subset minimization problem (where the parameter *k* still denotes the *size* of the desired optimal solution, we give additional details in the next paragraph), we obtain a β-approximation algorithm running in time $\text {\texttt {amls}}(\alpha ,c,\beta )^{n + o(n)}$. Note that this matches the corresponding bound in the unweighted setting up to subexponential factors.

To state our main result more precisely, let us formalize the requirements for the parameterized α-approximation algorithm. Similar to [3], we require an α*-approximate extension algorithm*. Such an algorithm receives as input a set X⊆U and a number k≥0. If there is an extension S⊆U to a solution set (i.e., $X \cup S \in {\mathcal {F}}$) of size at most *k*, then the algorithm outputs a set T⊆U such that $X \cup T \in {\mathcal {F}}$ and $\textbf{w}(T) \le \alpha \cdot \textbf{w}(S^*)$ where $S^*$ is a minimum-weight extension of *X* of size at most *k*. Note that the size of *T* does not need to be bounded in *k*; the parameter *k* only restricts the size of an “optimum solution” which we compare against. For example, the polynomial-time 2-approximation algorithm for Weighted Vertex Cover [2] immediately results in a 2-approximate extension algorithm: given a graph *G*, X⊆V(G) and k≥0, we apply the 2-approximation algorithm to G-X and return the output T⊆V(G) (this algorithm behaves independently of *k*). With this, our main result can informally be stated as follows.

**Figure:**
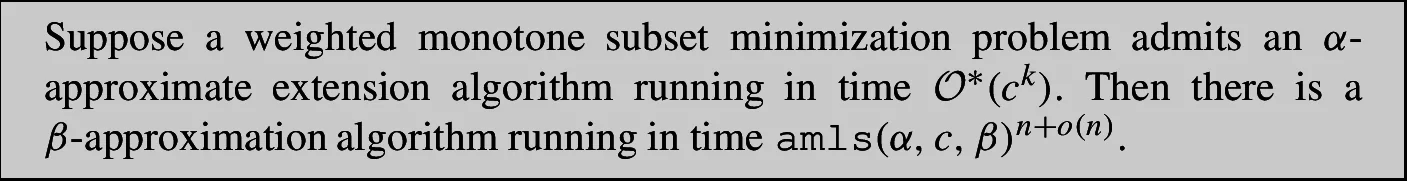

A formal version of our result can be found in Theorem 2.8.

The basic idea to achieve this result is to partition the universe *U* into subsets $U_i$ of elements of roughly the same weight. We apply the results from [3] to each of the sets $U_i$ separately which results in a “query set” for $U_i$, i.e., a set of queries made by the algorithm from [3] to the α-approximate extension algorithm. The crucial observation is that these queries are made in a non-adaptive way, i.e., the “query set” only depends on the set $U_i$. We then combine the “query sets” for the blocks $U_i$ into a query set for the whole weighted set *U*. In particular, the results from [3] are only used in a black-box manner.

For many problems such as Vertex Cover [2], *d*-Hitting Set [2] and Feedback Vertex Set [12], polynomial-time approximation algorithms directly extend to the weighted setting, and provide α-approximate extension algorithms as discussed above. On the other hand, while many parameterized exact algorithms do not directly extend to the weighted setting, several problems have been studied in the weighted setting. For example, for Weighted Vertex Cover [13] provides an $\mathcal {O}^*(1.363^k)$ algorithm that, given a vertex-weighted graph and a number k≥0, returns a vertex cover of weight at most *W* where *W* is the minimum weight of a vertex cover of size at most *k* (if there is no vertex cover of size at most *k*, the algorithm reports failure). As before, to obtain a 1-approximate extension subroutine, we apply the algorithm to the graph G-X (where *X* is the input set for the extension subroutine). Similar results are also available for Weighted *d*-Hitting Set [13, 14] and Weighted Feedback Vertex Set [15]. Also, some simple branching algorithms immediately extend to the weighted setting (e.g., deletion to $\mathcal {H}$-free graphs where $\mathcal {H}$ is a finite set of forbidden induced subgraphs). Finally, we can also rely on parameterized approximation algorithms. For example, [16] provides such algorithms for several problems including Weighted Directed FVS and Weighted Multicut.

We remark that there are also FPT algorithms for other parameterizations in the weighted setting. For example, Niedermeier and Rossmanith [17] show that Weighted Vertex Cover is fixed-parameter tractable parameterized by the weight *W* of a minimum-weight vertex cover. However, such subroutines are not useful in our setting since we aim to bound the running time of our approximation algorithms with respect to the number of vertices.

Similar to [3], we compare our algorithms to a brute-force search. Here, we consider a setting where the weighted monotone subset minimization problem can only be accessed via a membership oracle, i.e., given a set X⊆U, we can test (in polynomial time) whether *X* is a solution set. For every $\beta \ge 1$ we define $\text {\texttt {brute}}(\beta ) :=1 + \exp \left( -\beta \cdot {\mathcal {H}}\left( \frac{1}{\beta }\right) \right)$ where ${\mathcal {H}}(\beta ) :=-\beta \ln \beta - (1-\beta ) \ln (1-\beta )$ denotes the entropy function. In [4] it has been shown that, in the unweighted setting, there is a β-approximation algorithm running in time $(\text {\texttt {brute}}(\beta ))^{n+o(n)}$ that only exploits a membership test. We also extend this result to the weighted setting, i.e., we show that every weighted monotone subset minimization problem can be solved in time $(\text {\texttt {brute}}(\beta ))^{n + o(n)}$ given only a membership oracle.

Esmer et al. [3] show that $\text {\texttt {amls}}(\alpha ,c,\beta ) < \text {\texttt {brute}}(\beta )$ for all $\alpha ,c \ge 1$ and β>1. Since the same bounds are achieved in the weighted setting, all algorithms obtained above are strictly faster than the brute-force β-approximation algorithm. In particular, we obtain exponential-time approximation algorithms that are faster than the approximate brute-force search for the weighted versions of Vertex Cover, *d*-Hitting Set, Feedback Vertex Set, Tournament FVS, Subset FVS, Cluster Graph Vertex Deletion, Cograph Vertex Deletion, Split Vertex Deletion, Partial Vertex Cover, Directed Feedback Vertex Set, Directed Subset FVS, Directed Odd Cycle Transversal and Multicut (all problems are defined in Appendix D).

### Organization

The paper is organized as follows. In Sect. 2 we state the problems we want to address, provide the necessary definitions and notation, and formally state our main results. In Sect. 3, we demonstrate how our methods can be applied to specific problems to obtain exponential-time approximation algorithms. Sect. 4 contains the proof of Theorem 2.3, our result on exponential-time approximation algorithms for the $\text {WeightedSM}$ problem in the membership model. Similarly, Sect. 5 contains the proof of Theorem 2.8, our result on exponential-time approximation algorithms for the $\text {WeightedSM}$ problem in the extension model. Finally, in Sect. 6 we conclude the paper by summarizing our main contributions and key findings.

## Computational Model and Our Results

To formally state our results we use an abstract notion of a problem and oracle-based computational models. Let *U* be a finite set of elements. We use *n* to denote the cardinality of *U*. A set system ${\mathcal {F}}$ of *U* is a family ${\mathcal {F}}\subseteq 2^U$ of subsets of *U*. We say the set system ${\mathcal {F}}$ is *monotone* if (i) $U\in {\mathcal {F}}$ and (ii) for all $T\subseteq S\subseteq U$, if $T\in {\mathcal {F}}$ then $S\in {\mathcal {F}}$ as well.

An instance of the $\text {Weighted Monotone Subset Minimization}$ problem ($\text {WeightedSM}$ for short) is a triplet $(U,\textbf{w},{\mathcal {F}})$ where *U* is a finite set, $\textbf{w}:U\rightarrow \mathbb {N}$ is a weight function over the elements of *U*, and ${\mathcal {F}}$ is a monotone set system of *U*. The set of solutions is ${\mathcal {F}}$ and the objective is to find $S\in {\mathcal {F}}$ with minimum total weight $\textbf{w}(S) :=\sum _{u\in S} \textbf{w}(u)$. We use $\texttt {opt}(U,\textbf{w},{\mathcal {F}}) :=\min \{\textbf{w}(S) \mid S\in {\mathcal {F}}\}$ to denote the optimum value of a solution to the $\text {WeightedSM}$ instance $(U,\textbf{w},{\mathcal {F}})$. We refer to the special case in which $\textbf{w}(u)=1$ for all u∈U as the $\text {Unweighted Monotone Subset Minimization}$ ($\text {UnweightedSM}$).

$\text {WeightedSM}$ is a meta-problem which captures multiple well studied problems as special cases, e.g., Weighted Vertex Cover, Weighted Feedback Vertex Set and Weighted Multicut. We study the problem using two computational models: the *membership model* and the *extension model*. In both models the set *U* and the weight function $\textbf{w}$ are given as part of the input. The set ${\mathcal {F}}$ can only be accessed using an oracle, and the models differ in the type of supported oracle queries.

### Membership Oracles and Weighted Approximate Brute Force

In the *membership model* the input to the algorithm is a universe *U* and a weight function $\textbf{w}:U \rightarrow \mathbb {N}$. Additionally, the algorithm has access to a membership oracle for a monotone set system ${\mathcal {F}}$ of *U*, that is, the algorithm can check if a subset S⊆U satisfies $S\in {\mathcal {F}}$ in a single step. For every $\alpha \ge 1$, we say an algorithm is an α-approximation for $\text {WeightedSM}$ ($\text {UnweightedSM}$) in the membership model if for every $\text {WeightedSM}$ ($\text {UnweightedSM}$) instance $(U,\textbf{w},{\mathcal {F}})$ the algorithm returns a set $S\in {\mathcal {F}}$ such that $\textbf{w}(S)\le \alpha \cdot \texttt {opt}(U,\textbf{w},{\mathcal {F}})$.

One can easily attain a 1-approximation algorithm for $\text {WeightedSM}$ in the membership model by iterating over all subsets of *U* and querying the oracle for each. Since each oracle query is counted as a single step, this leads to an algorithm with running time $\mathcal {O}^*(2^{n})$. We refer to this algorithm as the (exact) brute force. Moreover, it can be shown by a simple adversarial argument that there is no 1-approximation algorithm for $\text {WeightedSM}$ (or for $\text {UnweightedSM}$) in the membership model which runs in time $\mathcal {O}\left( (2-\varepsilon )^{n}\right)$.

Intuitively, for every α>1, it should be possible to design an α-approximation algorithm for $\text {WeightedSM}$ and $\text {UnweightedSM}$ in the membership model which runs in time $\mathcal {O}(c^n)$ for some c<2. However, the smallest achievable *c* (i.e., the optimal value) in this setting is not evident. In [4] the authors studied $\text {UnweightedSM}$ in the membership model and pinpointed the right value of *c*. For every $\alpha \ge 1$ we define

1 $$\begin{aligned} \text {\texttt {brute}}(\alpha ) = 1+\exp \left( -\alpha \cdot {\mathcal {H}}\left( \frac{1}{\alpha } \right) \right) , \end{aligned}$$

where ${\mathcal {H}}(x) = -x\ln (x) -(1-x) \ln (1-x)$ is the entropy function.

#### Lemma 2.1

( [4, Theorem 5.1]) For every $\alpha \ge 1$ the following holds.

1.: There is a deterministic α-approximation algorithm for $\text {UnweightedSM}$ in the membership model which runs in time $\left( \text {\texttt {brute}}(\alpha )\right) ^{n+o(n)}$.^2^
2.: Let ε>0. There is no deterministic α-approximation algorithm for $\text {UnweightedSM}$ in the membership model which runs in time $\left( \text {\texttt {brute}}(\alpha )-\varepsilon \right) ^n\cdot n^{\mathcal {O}(1)}$.

As the algorithmic result in Lemma 2.1 can be viewed as an approximate analogue of the brute-force algorithm, it is commonly referred as α*-approximate brute force*. We note that the lower bound given in Lemma 2.1 also holds in the more general case where the algorithm is allowed to use randomization. We refer the reader to [4] for the definition of randomized approximation algorithms and the results in this setting. On the other hand, since $\text {WeightedSM}$ is a generalization of $\text {UnweightedSM}$, the following corollary is an immediate consequence of the lower bound in Lemma 2.1.

#### Corollary 2.2

For every $\alpha \ge 1$ and ε>0 there is no α-approximation algorithm for $\text {WeightedSM}$ in the membership model which runs in time $\left( \text {\texttt {brute}}(\alpha )-\varepsilon \right) ^n\cdot n^{\mathcal {O}(1)}$.

Our first result is a generalization of the approximate brute-force algorithm of [4] for the weighted setting. That is, we provide an algorithm which matches the lower bound in Corollary 2.2 up to a sub-exponential factor.

#### Theorem 2.3

(Weighted Approximate Brute Force) For every α>1 there is an α-approximation algorithm for $\text {WeightedSM}$ in the membership model which runs in time $\text {\texttt {brute}}(\alpha )^{n+o(n)}$.

The proof of Theorem 2.3 is based on a rounding of the weight function $\textbf{w}$ and utilizes a construction from [4] which is applied to each of the rounded weight classes.

### Extension Oracles and Weighted Approximate Monotone Local Search

Our second computational model deals with *extension oracles*. The input for these oracles is a set S⊆U and a number $\ell \in \mathbb {N}_{\ge 0}$ and the output is an *extension* of *S*, that is, a set X⊆U such that $S\cup X \in {\mathcal {F}}$. Furthermore, the returned set *X* is guaranteed to have a small weight in comparison to the minimum-weight extension of *S* which contains at most ℓ elements. For multiple problems, such as Vertex Cover and Feedback Vertex Set, these oracles can be implemented using existing parameterized algorithms which have a running time of the form $c^{\ell } \cdot n^{\mathcal {O}(1)}$. We therefore associate a running time of $c^{\ell }$ with the query (S,ℓ). The formal definition of extension oracles is as follows.

#### Definition 2.4

*(Extension Oracle)* Let $(U,\textbf{w},{\mathcal {F}})$ be a $\text {WeightedSM}$ instance and let $\alpha \ge 1$. An α-extension oracle for $(U,\textbf{w},{\mathcal {F}})$ is a function $\text {\texttt {Ext}}:2^U\times \mathbb {N}_{\ge 0} \rightarrow 2^U$ such that for every S⊆U and $\ell \in \mathbb {N}_{\ge 0}$ the following holds:

1. $$\text {\texttt {Ext}}(S,\ell ) \cup S\in {\mathcal {F}}$$.
2. $$\textbf{w}\left( \text {\texttt {Ext}}(S,\ell )\right) \le \alpha \cdot \min \{ \textbf{w}(X) \mid X\subseteq U,~{\left| X\right| }\le \ell ,~X\cup S\in {\mathcal {F}}\}$$ (we set $$\min \emptyset =\infty $$).

In the (α,c)*-extension model* the input for the algorithm is a finite set *U* and a weight function $\textbf{w}:U\rightarrow \mathbb {N}$. Furthermore, the algorithm is given oracle access to an α-extension oracle $\text {\texttt {Ext}}$ of $(U,\textbf{w},{\mathcal {F}})$ for some monotone set system ${\mathcal {F}}$ of *U*. For every $\beta \ge 1$, we say an algorithm is a β-approximation algorithm for $\text {WeightedSM}$ ($\text {UnweightedSM}$) in the (α,c)-extension model if for every $\text {WeightedSM}$ ($\text {UnweightedSM}$) instance $(U,\textbf{w},{\mathcal {F}})$ and α-extension oracle $\text {\texttt {Ext}}$ of the instance, the algorithm returns $T\in {\mathcal {F}}$ such that $\textbf{w}(T)\le \beta \cdot \texttt {opt}(U,\textbf{w},{\mathcal {F}})$. The running time of an algorithm in this model is the number of computation steps plus $c^\ell$ for every query (S,ℓ) issued to the oracle during the execution. Following the standard worst-case analysis convention, we say an algorithm runs in time *f*(*n*) if for every $\text {WeightedSM}$ instance $(U,\textbf{w},{\mathcal {F}})$ and α-extension oracle $\text {\texttt {Ext}}$ of the instance the algorithm runs in time at most $f\left( {\left| U\right| }\right)$.

The (α,c)-extension model has been studied in [3] for the special case of $\text {UnweightedSM}$. The authors of [3] provide a deterministic β-approximation algorithm in the (α,c)-extension model, known as *deterministic approximate monotone local search*, which runs in time $\left( \text {\texttt {amls}}(\alpha ,c,\beta )\right) ^{n+o(n)}$, where $\text {\texttt {amls}}(\alpha ,c,\beta )$ is defined as the optimal value of a continuous optimization problem. Throughout the paper we use $\text {\texttt {amls}}$ to denote this function. We provide the formal definition of $\text {\texttt {amls}}$ in Appendix B for completeness. We note this formal definition is not required for the understanding of the results in this paper. It is also shown in [3] that the value of $\text {\texttt {amls}}(\alpha ,c,\beta )$ can be computed up to precision of ε in time polynomial in the encoding length of α, *c*, β and ε.

This algorithmic result is complemented in [3] with a matching lower bound.

#### Lemma 2.5

( [3]) For every $\alpha ,\beta ,c\ge 1$ and ε>0 there is no deterministic β-approximation for $\text {UnweightedSM}$ in the (α,c)-extension model which runs in time $\left( \text {\texttt {amls}}(\alpha ,c,\beta )-\varepsilon \right) ^{n} \cdot n^{\mathcal {O}(1)}$.

Furthermore, it is shown that the running time of deterministic approximate monotone local search is better than brute force for β>1.

#### Lemma 2.6

( [3]) For all $\alpha ,c\ge 1$ and β>1 it holds that $\text {\texttt {amls}}(\alpha ,c,\beta ) <\text {\texttt {brute}}(\beta )$.

The results of [3] also include a randomized algorithm which omits the subexponential factor in the running time and the lower bound also holds for randomized algorithms. In [3] the authors use the deterministic approximate monotone local search algorithm to obtain exponential-time approximation algorithms for multiple *unweighted* problems such as Vertex Cover and Feedback Vertex Set. We generalize the algorithmic results of [3] to the weighted setting and similarly use it to obtain exponential-time approximation algorithms for *weighted* variants of the mentioned problems (see Sect. 3).

Since $\text {UnweightedSM}$ is a special case of $\text {WeightedSM}$, Lemma 2.5 immediately implies the following.

#### Corollary 2.7

For every $\alpha ,c,\beta \ge 1$ and ε>0 there is no deterministic β-approximation for $\text {WeightedSM}$ in the (α,c)-extension model which runs in time $\left( \text {\texttt {amls}}(\alpha ,c,\beta )-\varepsilon \right) ^{n} \cdot n^{\mathcal {O}(1)}$.

Our second result is an algorithm which matches the running time in Corollary 2.7 up to subexponential factors.

#### Theorem 2.8

(Weighted Approximate Monotone Local Search) For every $\alpha ,c\ge 1$, β>1 there is a deterministic β-approximation for $\text {WeightedSM}$ in the (α,c)-extension model which runs in time $\text {\texttt {amls}}(\alpha ,c,\beta )^{n + o(n)}$.

The proof of Theorem 2.8 is similar to the one of Theorem 2.3. It applies rounding to the weights on the elements, and then utilizes a construction from [3] for each of the weight classes.

The result of Theorem 2.8 only applies for β>1. If $\alpha =\beta =1$ the result of [11] can be used to obtain an exact algorithm with running time $n^{\mathcal {O}(1)}\cdot \left( 2-\frac{1}{c}\right) ^n$. For $\alpha >\beta =1$ Corollary 2.7 implies that the best possible running time is $\mathcal {O}^*(2^{n})$ which can be attained by brute force.

## Applications

Our results provide exponential-time approximation algorithms for a variety of weighted vertex-deletion problems. For illustration purposes, let us first focus on the Weighted Vertex Cover problem where we are given a graph *G* with vertex weights $\textbf{w}:V(G) \rightarrow \mathbb {N}$, and we ask for a vertex cover S⊆V(G) of minimum weight $\textbf{w}(S) = \sum _{v \in S} \textbf{w}(v)$. It is well-known that Weighted Vertex Cover admits a polynomial-time 2-approximation algorithm [2]. Also, the problem can be solved exactly in time $\mathcal {O}^*(1.238^n)$ [18].

For the unweighted version Unweighted Vertex Cover, Bourgeois, Escoffier and Paschos [7] designed several exponential-time approximation algorithms for approximation ratios in the range (1, 2). For example, they obtain a 1.1-approximation algorithm running in time $\mathcal {O}^*(1.127^n)$ where *n* denotes the number of vertices of the input graph. These running times are further improved in [3, 4] using the framework of approximate monotone local search. Indeed, the fastest known 1.1-approximation algorithm for Unweighted Vertex Cover runs in time $\mathcal {O}^*(1.113^n)$ [3].

**Fig. 1:**
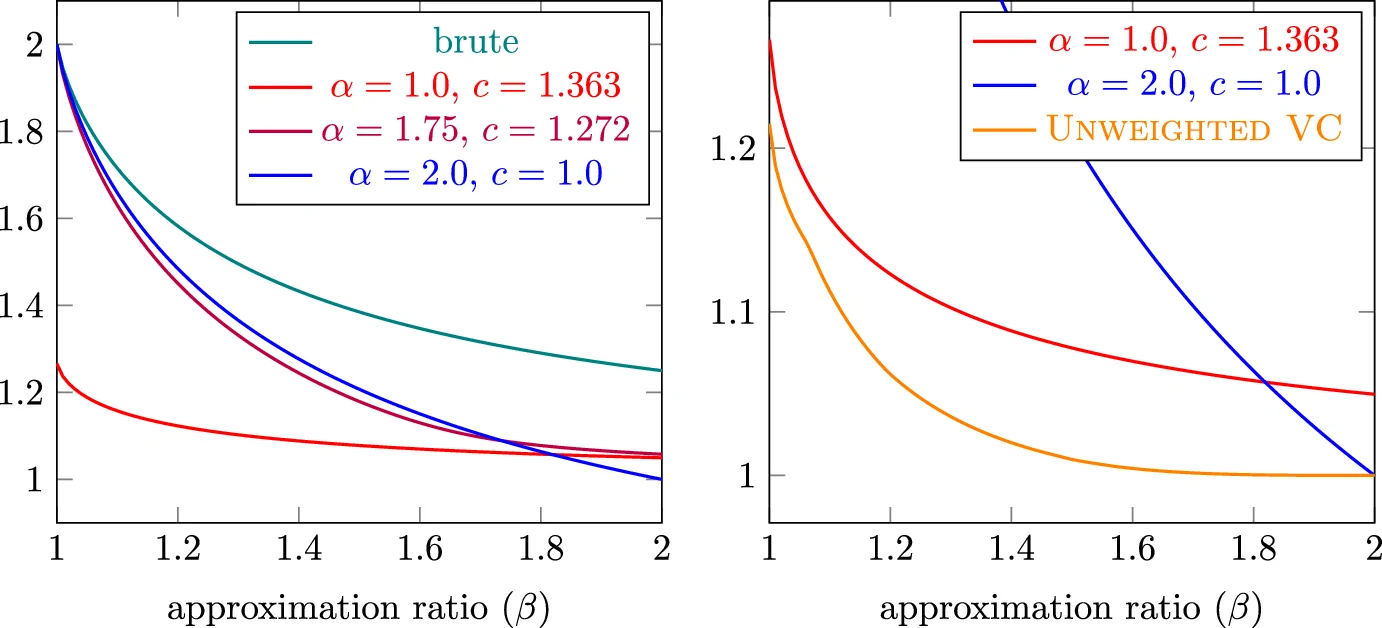
Running times for β-approximation algorithms for Weighted Vertex Cover obtained via Theorem 2.8. For each curve and approximation ratio β, the plotted value *d* denotes the base in the running time $d^{n+o(n)}$ of the corresponding β-approximation algorithm. The left plot compares brute-force baseline against the algorithms obtained by using extension oracles with parameters $(\alpha ,c) \in \{ ( 1.0, 1.363), ( 1.75, 1.272), (2.0, 1.0)\}$. The right plot compares the algorithms obtained by using oracles $(\alpha ,c) \in \{ ( 1.0, 1.363), (2.0, 1.0)\}$ against the best known running times for Unweighted Vertex Cover from [3]

For the weighted version, no exponential-time approximation algorithms have been obtained so far. We use Theorem 2.8 to design the first exponential β-approximation algorithms for Weighted Vertex Cover for all $\beta \in (1,2)$. For the extension oracle, we use the well-known polynomial-time 2-approximation algorithm. Given a set S⊆V(G), we delete all vertices in *S* and apply the 2-approximation algorithm to the graph G-S which outputs a vertex cover *X* such that $\textbf{w}(X) \le 2 \cdot \textbf{w}(\texttt {OPT})$ where $\texttt {OPT}$ denotes a minimum vertex cover of G-S. As a result, we can implement a 2-extension oracle in polynomial time which corresponds (up to polynomial factors) to cost c=1. So Theorem 2.8 results in a β-approximation algorithm for Weighted Vertex Cover which runs in time $\text {\texttt {amls}}(\alpha ,c,\beta )^{n+o(n)}$ for every β>1, where α=2 and c=1. A visualization is given in Figure 1.

Instead of using a polynomial-time 2-approximation algorithm, we can also rely on existing FPT algorithms for Weighted Vertex Cover to simulate the extension oracle. Let us point out that different parameterizations have been considered for Weighted Vertex Cover in the literature. For example, Niedermeier and Rossmanith [17] give FPT algorithms for Weighted Vertex Cover parameterized by the weight of the optimal solution. However, in light of the computational model introduced above, we require FPT algorithms parameterized by the *size* of the solution. Given a graph *G* with vertex weights $\textbf{w}:V(G) \rightarrow \mathbb {N}$ and an integer k≥0, we ask for a vertex cover of weight at most *W* where *W* is the minimum weight of a vertex cover of size at most *k*. The best known FPT algorithm (parameterized by *k*) for this problem runs in time $1.363^k \cdot n^{\mathcal {O}(1)}$ [13]. This algorithm provides an extension algorithm with parameters α=1 and c=1.363. Using Theorem 2.8, we obtain a β-approximation algorithm for Weighted Vertex Cover running in time $\text {\texttt {amls}}(\alpha ,c,\beta )^{n+o(n)}$. For β=1.1, we obtain a running time of $1.158^{n+o(n)}$.

Moreover, we can also consider parameterized approximation algorithms. More precisely, for $\alpha \ge 1$, we can use an FPT approximation algorithm that is parameterized by the solution size, and approximates the optimal weight (among all solutions of size at most *k*) up to a factor of α. Formally, given a graph *G* with vertex weights $\textbf{w}:V(G) \rightarrow \mathbb {N}$ and an integer k≥0, we ask for a vertex cover of weight at most $\alpha \cdot W$ where *W* is the minimum weight of a vertex cover of size at most *k*. Mandal, Rai and Saurabh [19] design such algorithms for various approximation ratios α. For example, for α=1.75, they obtain an algorithm running time $1.272^k \cdot n^{\mathcal {O}(1)}$, which provides an extension algorithm with parameters α=1.75 and c=1.272. Unfortunately, using an algorithm from [19] as an extension subroutine always leads to a slower exponential-time approximation algorithm compared to the two extension algorithms described above. One reason is that [19] actually considers a more general problem (where arbitrary rational weights are allowed).

**Table 1:** The table shows the running times for Weighted Vertex Cover (second and third row) and Unweighted Vertex Cover (last row). An entry *d* in column β means that the respective algorithm outputs a β-approximation in time $d^{n + o(n)}$.

	1.1	1.2	1.3	1.4	1.5	1.6	1.7	1.8	1.9
$\text {\texttt {brute}}$	1.716	1.583	1.496	1.433	1.385	1.347	1.317	1.291	1.269
(α=1,c=1.363)	1.158	1.123	1.103	1.089	1.078	1.07	1.064	1.058	1.054
(α=1.75,c=1.272)	1.631	1.451	1.332	1.245	1.18	1.13	1.097	1.078	1.067
(α=2,c=1)	1.659	1.485	1.366	1.277	1.208	1.151	1.104	1.064	1.03
Unweighted VC	1.113	1.062	1.036	1.02	1.01	1.005	1.002	1.0004	1.00005

We provide running times for selected approximation ratios for all three algorithms in Table 1 and a graphical comparison in Figure 1. We also compare the running times to the approximate brute-force search and the best algorithms in the unweighted setting [3]. It can be observed that the second algorithm (using the FPT algorithm as an extension subroutine) is fastest for $\beta \lesssim 1.82$; for $\beta \gtrsim 1.82$ the first algorithm (using the polynomial-time 2-approximation algorithm as an extension subroutine) is fastest. Also, there is still a noticeable gap to the unweighted setting.

**Table 2:** List of weighted deletion problems admitting a single-exponential parameterized algorithm running in time $O^*(c_1^k)$ and/or a polynomial-time $\alpha _2$-approximation algorithm. The problems Tournament FVS, Cluster Graph Vertex Deletion, Cograph Vertex Deletion and Split Vertex Deletion can be easily reduced to *d*-Hitting Set for appropriate values of *d* by exploiting known characterizations in terms of forbidden induced subgraphs. Additionally, for Tournament FVS we can rely on iterative compression to obtain a $\mathcal {O}^*(2^k)$ algorithm (see, e.g., [22]).

Problem	$c_1$		det.	$\alpha _2$		det.
Vertex Cover	1.363	[13]	✓	2	[2]	✓
FVS	3.618	[15]	✓	2	[12]	✓
Tournament FVS	2.0		✓	3		✓
Subset FVS	-		✓	8	[20]	✓
3-Hitting Set	2.168	[13]	✓	3	[2]	✓
*d*-Hitting Set (d≥4)	d-0.832	[14]	✓	*d*	[2]	✓
Cluster Graph Vertex Deletion	2.168		✓	3		✓
Cograph Vertex Deletion	3.168		✓	4		✓
Split Vertex Deletion	4.168		✓	5		✓
Partial Vertex Cover	-		✓	2	[21]	✓

**Fig. 2:**
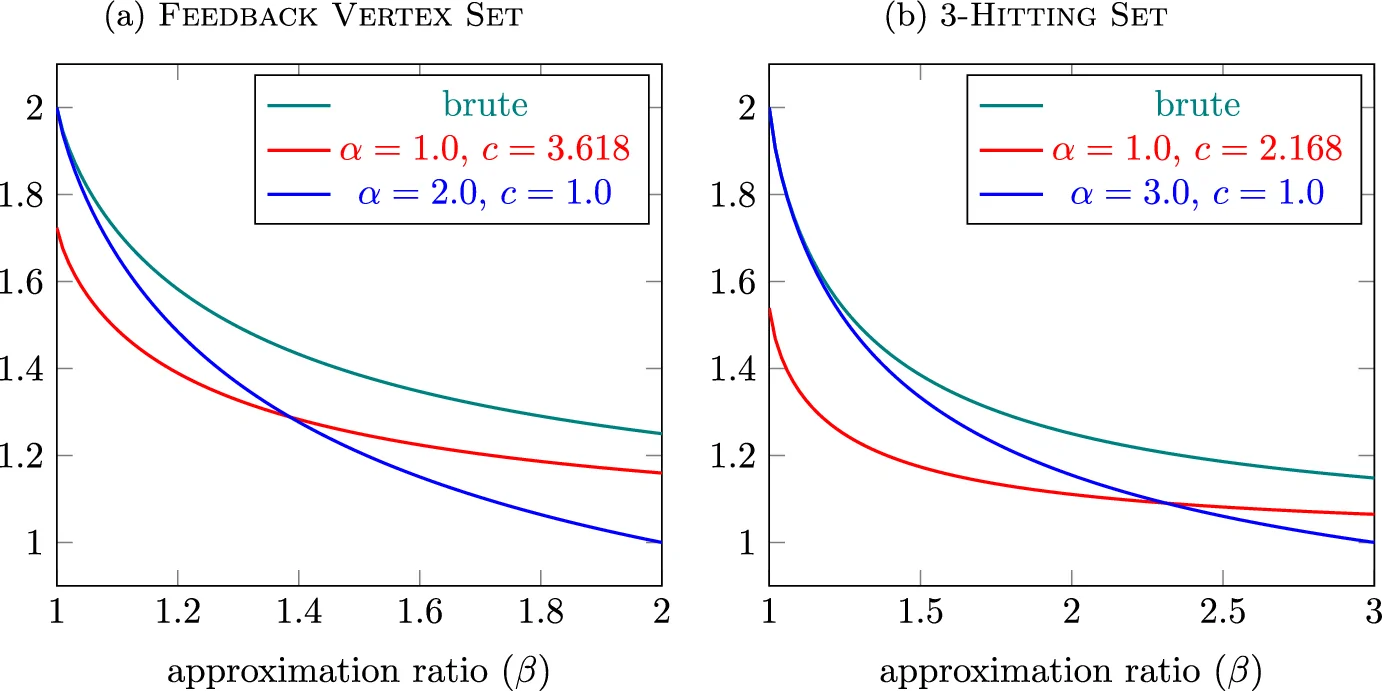
The left plot shows running times for Feedback Vertex Set, and the right plot shows running times for 3-Hitting Set. For each curve and approximation ratio β, the plotted value *d* denotes the base in the running time $d^{n + o(n)}$ of the corresponding β-approximation algorithm

We stress that our results are not limited to Weighted Vertex Cover, but they are applicable to various vertex-deletion problems including Weighted Feedback Vertex Set and Weighted *d*-Hitting Set (see Figure 2). Table 2 gives an overview on problems for which we obtain exponential approximation algorithms by simulating the extension oracle by an FPT algorithm (parameterized by solution size) running in time $\mathcal {O}^*(c_1^k)$ or a polynomial-time $\alpha _2$-approximation algorithm. For all the problems listed in Table 2, we obtain the first exponential β-approximation algorithms for all $\beta \in (1,\alpha _2)$. Observe that these algorithms always outperform the approximate brute-force search by Lemma 2.6. We provide data on the running times of these algorithms in Appendix 7.

Finally, several parameterized approximation algorithms for a weighted vertex deletion problems are obtained in [16], namely for Weighted Directed Feedback Vertex Set, Weighted Directed Subset FVS, Weighted Directed Odd Cycle Transversal and Weighted Multicut. As a result, we also obtain exponential β-approximation algorithms for these problems that outperform the approximate brute-force search.

## Weighted Approximate Brute Force

In this section we prove Theorem 2.3. The algorithm is based on the notion of *covering families*.

### Definition 4.1

(Covering Family) Let *U* be a finite set, $\textbf{w}:U\rightarrow \mathbb {N}$ be a weight function and $\alpha \ge 1$. We say ${\mathcal {C}}\subseteq 2^U$ is an α*-covering family* of *U* and $\textbf{w}$ if for every S⊆U there exists $T\in {\mathcal {C}}$ such that S⊆T and $\textbf{w}(T)\le \alpha \cdot \textbf{w}(S)$.

An α-covering family ${\mathcal {C}}$ can be easily used to attain an α-approximation algorithm in the membership model as follows. The algorithm constructs the covering family ${\mathcal {C}}$ and uses the membership oracle to compute ${\mathcal {C}}\cap {\mathcal {F}}$ using ${\left| {\mathcal {C}}\right| }$ queries. The algorithm then returns the set $Q\in {\mathcal {C}}\cap {\mathcal {F}}$ of minimum weight. To show correctness, consider a set $S\in {\mathcal {F}}$ such that $\textbf{w}(S)= \texttt {opt}(U,\textbf{w},{\mathcal {F}})$. Since ${\mathcal {C}}$ is an α-covering family there is $T\in {\mathcal {C}}$ such that S⊆T and $\textbf{w}(T)\le \alpha \cdot \textbf{w}(S)= \alpha \cdot \texttt {opt}(U,\textbf{w},{\mathcal {F}})$. Since ${\mathcal {F}}$ is monotone it holds that $T\in {\mathcal {F}}$, thus $T\in {\mathcal {C}}\cap {\mathcal {F}}$, and we conclude that the algorithm returns a set of weight at most $\alpha \cdot \texttt {opt}(U,\textbf{w},{\mathcal {F}})$. The running time of the algorithm, up to polynomial factors, is the construction time of ${\mathcal {C}}$ plus ${\left| {\mathcal {C}}\right| }$.

By the above argument, the proof of Theorem 2.3 boils down to the construction of α-covering families. The next result provides this construction. Recall that *n* denotes the size of the universe *U*.

### Lemma 4.2

There exists an algorithm which given a finite set *U*, a weight function $\textbf{w}:U\rightarrow \mathbb {N}$ and α>1, constructs an α-covering family ${\mathcal {C}}$ of *U* and $\textbf{w}$ such that ${\left| {\mathcal {C}}\right| } \le \left( \text {\texttt {brute}}(\alpha )\right) ^{n+o(n)}$. Furthermore, the running time of the algorithm is $\left( \text {\texttt {brute}}(\alpha )\right) ^{n+o(n)}$.

By the argument above, Theorem 2.3 follows directly from Lemma 4.2. We therefore devote the rest of this section to proving Lemma 4.2. The construction of the covering family in Lemma 4.2 is based on a rounding of the weight function and a reduction to covering families in the unweighted case. In the unweighted setting, the following lemma from [4] provides an implicit construction of such families.

### Lemma 4.3

( [4]) There exists an algorithm which given a finite set *U* and α>1 returns an α-covering family ${\mathcal {C}}$ of *U* and the uniform weight function $\textbf{w}:U\rightarrow \{1\}$ such that ${\left| {\mathcal {C}}\right| }\le \left( \text {\texttt {brute}}(\alpha )\right) ^{n+o(n)}$. Furthermore, the algorithm runs in time $\left( \text {\texttt {brute}}(\alpha )\right) ^{n+o(n)}$.

Moreover, we need the following technical lemma whose proof is given in Appendix A.

### Lemma 4.4

Let $g,d:\mathbb {N}\rightarrow \mathbb {N}$ be two functions such that g∈n+o(n) and d∈o(n). We define $f:\mathbb {N}\rightarrow \mathbb {N}$ via

$$\begin{aligned} f(n) = \max _{n = n_1 + \dots + n_{d(n)}} \sum _{i=1}^{d(n)} g(n_i). \end{aligned}$$

Then f∈n+o(n).

We first prove a weaker version of Lemma 4.2.

### Lemma 4.5

Let *U* be a given finite set with $n :={\left| U\right| }$, $\textbf{w}:U\rightarrow \mathbb {N}$ be a weight function, β>1 and $\delta :\mathbb {N}\rightarrow (0,1]$ be a function such that $\delta (n) = \Omega \left( 1 / \log (n)\right)$. Then, there exists a deterministic algorithm $\mathscr {A}$ that constructs a $((1 + \delta (n)) \cdot \beta )$-covering family ${\mathcal {C}}$ of *U* and $\textbf{w}$ such that ${\left| {\mathcal {C}}\right| } \le \left( \text {\texttt {brute}}(\beta )\right) ^{n + o(n)}$. Furthermore, the running time of the algorithm is $\left( \text {\texttt {brute}}(\beta )\right) ^{n + o(n)}$.

### Proof

In the proof, γ,d (which are defined later) and δ are functions of *n*, but we omit the dependence on *n* for the sake of readability. The algorithm $\mathscr {A}$ works as follows:

- Define $\gamma :=1 + \frac{\delta }{2} > 1$. For i≥0 let
  2 $$\begin{aligned} U_i :=\{u \in U \mid \gamma ^i \le \textbf{w}(u) < \gamma ^{i+1}\} \end{aligned}$$
  and $n_i :=|U_i|$. Let $I :=\{i \in {\mathbb {Z}}_{\ge 0} \mid U_i \ne \emptyset \}$ denote the set of indices i≥0 for which $U_i$ is non-empty. Note that |I|≤n.
- Using Lemma 4.3, for each i∈I construct a β-covering family ${\mathcal {C}}_i$ of the universe $U_i$ and the uniform weight function $\textbf{w}:U_i \rightarrow \{1\}$.
- Define $d :=\lceil (2/\delta ) \cdot \log (2n/\delta )\rceil$ and for each k∈I, let $I_k :=\{i \in I \mid k-d \le i \le k\}$ denote the indices in *I* between k-d and *k*.
- For every k∈I, let $r_k :={\left| I_k\right| }$ and define
  $$\begin{aligned} W_k&:=\bigcup _{i \in \;I :0 \le i < k - d } U_i \qquad \text {and} \\ \mathcal {Q}_k&:=\left\{ W_k \cup E_1 \cup \ldots \cup E_{r_{k}} \;\Bigg | \; \left( E_1, \ldots , E_{r_k} \right) \in \prod _{i \in I_k} {\mathcal {C}}_i \right\} , \end{aligned}$$
  where $\Pi _{i\in I_k} {\mathcal {C}}_i$ denotes the Cartesian product of the sets ${\mathcal {C}}_i$ for $i\in I_k$.
- Return the set ${\mathcal {C}}:=\{\emptyset \}\cup \bigcup _{k \in I} \mathcal {Q}_k$.

Intuitively, the collection $\mathcal {Q}_k$ forms a covering family for all sets S⊆U whose highest weight class i∈I with $S \cap U_i \ne \emptyset$ is equal to *k*. Since the total weight of the items in $W_k$ is negligible compared to $\textbf{w}(S)$, we can include these items in every set of the covering family without significantly increasing its weight. In addition to $W_k$, each set in $\mathcal {Q}_k$ contains a set $E_i \in \mathcal {C}_i$ for every $i \in I_k$, which are used to cover $S \cap U_i$. In the following, we prove the lemma by first showing that $\mathscr {A}$ returns a $(1+\delta )\cdot \beta$-covering family. We then bound the size of the family ${\mathcal {C}}$ and the running time of $\mathscr {A}$, which completes the proof.

### Claim 4.6

The algorithm $\mathscr {A}$ returns a $((1 + \delta ) \cdot \beta )$-covering family of *U* and $\textbf{w}$.

### Proof

Let us pick a set S⊆U. We show that there exists $T \in {\mathcal {C}}$ such that S⊆T and $\textbf{w}(T) \le (1 + \delta ) \cdot \beta \cdot \textbf{w}(S)$. If S=∅, then we choose T=∅ which satisfies $\textbf{w}(S) = 0 = \textbf{w}(T)$. Therefore in the following let us assume that $S \ne \emptyset$.

By the definition of γ and *d*, it holds that

3 $$\begin{aligned} \gamma ^d \ge \left( 1 + \frac{\delta }{2}\right) ^{(2/\delta ) \cdot \log (2n/\delta )} \ge 2^{\log (2n/\delta )} = \frac{2n}{\delta } \end{aligned}$$

using that $(1 + \frac{1}{x})^x \ge 2$ for all x≥1. Let k∈I be the largest index such that $S \cap U_k \ne \emptyset$. It holds that

4 $$\begin{aligned} \textbf{w}(W_k) < n \cdot \gamma ^{k - d} \le \frac{\delta }{2} \cdot \gamma ^{k} \le \frac{\delta }{2} \cdot \textbf{w}(S) \end{aligned}$$

where the first inequality follows from the fact that ${\left| W_k\right| } \le n$ and each $u \in W_k$ belongs to a set $U_i$ where i≤k-d-1 and therefore $\textbf{w}(u) < \gamma ^{k - d - 1 + 1} = \gamma ^{k - d}$ by (2). The second inequality follows from (3) and finally the last inequality holds because by definition of *k*, there exists $u \in S \cap U_k$ such that $\textbf{w}(u) \ge \gamma ^{k}$ by (2).

For every $i \in I_k$ define $S_i :=S \cap U_i$. Since ${\mathcal {C}}_i$ is a β-covering family of $U_i$ and the *uniform weight function*, for each $i \in I_k$ there exists $T_i \in {\mathcal {C}}_i$ such that $S_i \subseteq T_i$ and ${\left| T_i\right| } \le \beta \cdot {\left| S_i\right| }$. Hence, for all $i \in I_k$ it holds that

5 $$\begin{aligned} \textbf{w}(T_i) \le \gamma ^{i + 1} \cdot {\left| T_i\right| } \le \gamma ^{i + 1} \cdot \beta \cdot {\left| S_i\right| } \le \gamma \cdot \beta \cdot \textbf{w}(S_i) \end{aligned}$$

where the first inequality follows from the fact that $T_i \subseteq U_i$ and (2), the second inequality follows from the definition of $T_i$ and finally the last one again follows from the fact that $S_i \subseteq U_i$ and (2).

Let $\overline{T} :=\bigcup _{i \in I_k} T_i$ and define

$$\begin{aligned} T :=\biggl (W_k \cup \overline{T}\biggr ) \in \mathcal {Q}_k \subseteq {\mathcal {C}}. \end{aligned}$$

Then we have

$$\begin{aligned} S = S \cap U = S \cap \left( \bigcup _{i \in I} U_i \right)&= \biggl (S \cap \Bigl ( \cup _{i \in I : i < k - d} U_i\Bigr ) \biggr ) \cup \biggl (S \cap \Bigl ( \cup _{i \in I_k} U_i \Bigr ) \biggr )\\&= \left( S \cap W_k \right) \cup \left( \bigcup _{i \in I_k} \left( S\cap U_i\right) \right) \\&\subseteq W_k \cup \left( \bigcup _{i \in I_k} T_i\right) \\&= T. \end{aligned}$$

Finally, it also holds that

$$\begin{aligned} \textbf{w}(T)&= \textbf{w}(W_k) + \sum _{i \in I_k} \textbf{w}(T_i) \\&\le \frac{\delta }{2} \cdot \textbf{w}(S) + \sum _{i \in I_k} \gamma \cdot \beta \cdot \textbf{w}(S_i)  &   \text {by } (4) \text { and } (5) \\&\le \frac{\delta }{2} \cdot \beta \cdot \textbf{w}(S) + \gamma \cdot \beta \cdot \textbf{w}(S)\\&\le (1 + \delta ) \cdot \beta \cdot \textbf{w}(S). \end{aligned}$$

This shows that ${\mathcal {C}}$ is a $(1 + \delta )\cdot \beta$-covering family of *U* and $\textbf{w}$. □

### Claim 4.7

$$\begin{aligned} {\left| {\mathcal {C}}\right| } \le \left( \text {\texttt {brute}}(\beta )\right) ^{n + o(n)}. \end{aligned}$$

### Proof

By Lemma 4.3, for each i∈I it holds that

6 $$\begin{aligned} {\left| {\mathcal {C}}_i\right| } \le \left( \text {\texttt {brute}}(\beta )\right) ^{n_i + o(n_i)}. \end{aligned}$$

Moreover, for every k∈I, we have

7 $$\begin{aligned} \begin{aligned} {\left| I_k\right| } \le d&\le c \cdot \frac{1}{\delta (n)} \cdot \log \left( \frac{n}{\delta (n)} \right) \\&\le c' \cdot \log (n) \cdot \log \left( n \cdot \log (n) \right) \\&= o(n) \end{aligned} \end{aligned}$$

where *c* and $c^{\prime }$ are constants, and the second inequality hold because $$\delta (n) = \Omega \left( \frac{1}{\log (n)} \right) $$. Furthermore,

$$\begin{aligned} {\left| \mathcal {Q}_k\right| } \le {\left| \prod _{i \in I_k} {\mathcal {C}}_i\right| } = \prod _{i \in I_k} {\left| {\mathcal {C}}_i\right| }&\le \prod _{i \in I_k} \left( \text {\texttt {brute}}(\beta )\right) ^{n_i + o(n_i)}\\&= \left( \text {\texttt {brute}}(\beta )\right) ^{\sum _{i \in I_k} \left( n_i + o(n_i) \right) }\\&= \left( \text {\texttt {brute}}(\beta )\right) ^{n + o(n)}, \end{aligned}$$

where the last equality follows from Lemma 4.4 and (7). Finally, we have that

$$\begin{aligned} {\left| {\mathcal {C}}\right| } \le \Biggl (1+\biggl | \bigcup _{k \in I} \mathcal {Q}_k \biggr |\Biggr ) \le \Biggl (1+\sum _{k \in I} {\left| \mathcal {Q}_k\right| }\Biggr ) = \left( \text {\texttt {brute}}(\beta )\right) ^{n + o(n)} \end{aligned}$$

since $${\left| I\right| } \le n$$. □

### Claim 4.8

The running time of $\mathscr {A}$ is $\left( \text {\texttt {brute}}(\beta )\right) ^{n + o(n)}$.

### Proof

The construction of $\{{\mathcal {C}}_i\}_{i \in I}$ takes

8 $$\begin{aligned} \sum _{i \in I} \left( \text {\texttt {brute}}(\beta )\right) ^{n_i + o(n_i)} \le \left( \text {\texttt {brute}}(\beta )\right) ^{n + o(n)} \end{aligned}$$

which follows from Lemma 4.3 and the fact that $n_i \le n$ for all i∈I. Finally, the construction of ${\mathcal {C}}$ takes time proportional to the size of ${\mathcal {C}}$ where we have

$$\begin{aligned} {\left| {\mathcal {C}}\right| } = \left( \text {\texttt {brute}}(\beta )\right) ^{n + o(n)} \end{aligned}$$

by Claim 4.7. All in all, the running time of $\mathscr {A}$ is upper bounded by

$$\begin{aligned} \left( \text {\texttt {brute}}(\beta )\right) ^{n + o(n)} . \end{aligned}$$

□

The algorithm $\mathscr {A}$ returns a $((1 + \delta (n)) \cdot \beta )$-covering family by Claim 4.6. Moreover, the size of ${\mathcal {C}}$ is at most $\left( \text {\texttt {brute}}(\beta )\right) ^{n + o(n)}$ by Claim 4.7, and the running time of $\mathcal {A}$ is $\left( \text {\texttt {brute}}(\beta )\right) ^{n + o(n)}$ by Claim 4.8. □

To complete the proof of Lemma 4.2 we use Lemma 4.5 together with the following technical lemma.

### Lemma 4.9

Let $f :I \rightarrow (1,\infty )$ be a continuous function on an open interval $I\subseteq \mathbb {R}$, $\alpha \in I$ with α>1 and let $g :\mathbb {N}\rightarrow \mathbb {N}$ such that g∈o(n). Define $\beta (n) :=\alpha - \frac{1}{\log (n)}$ for all $n \in \mathbb {N}$. Then it holds that

$$\begin{aligned} f\bigl (\beta (n)\bigr )^{n + g(n)} = f(\alpha )^{n + o(n)}. \end{aligned}$$

The proof of Lemma 4.9 is given in Appendix C.

### Proof of Lemma 4.2

We claim that the algorithm $\mathscr {A}$ from Lemma 4.5 with $\beta :=\alpha - \frac{1}{\log (n)}$ and $\delta :=\frac{\alpha }{\beta } - 1$ satisfies the properties listed in Lemma 4.2. Note that β and δ are functions of *n*, but we write β and δ instead of β(n) and δ(n) for the sake of readability.

Observe that we have $(1 + \delta ) \cdot \beta = \frac{\alpha }{\beta } \cdot \beta = \alpha$. Hence, by Lemma 4.5, there exists a function $g :\mathbb {N} \rightarrow \mathbb {N}$ such that g∈o(n), and the set returned by $\mathscr {A}$ is an α-covering family ${\mathcal {C}}$ of *U* and $\textbf{w}$ where ${\left| {\mathcal {C}}\right| } \le \left( \text {\texttt {brute}}(\beta )\right) ^{n + g(n)}$. The running time of the algorithm is also bounded by $\left( \text {\texttt {brute}}(\beta )\right) ^{n + g(n)}$.

By (1), $\text {\texttt {brute}}(x)$ is a continuous function of *x* because the entropy function is continuous. Therefore by Lemma 4.9 it holds that

$$\begin{aligned} \left( \text {\texttt {brute}}(\beta )\right) ^{n + g(n)} = \left( \text {\texttt {brute}}(\alpha )\right) ^{n + o(n)} \end{aligned}$$

which proves the lemma. □

## Weighted Monotone Local Search

In this section we prove Theorem 2.8. The proof presented here follows the outline of the proof of Theorem 2.3 in Sect. 4, using the concept of an $(\alpha ,\beta )$*-extension family*, an adaptation of the term α-covering family presented in Sect. 4 to the setting of extension oracles. Recall that the items in a covering family are queries to a membership oracle, and hence are subsets of *U*. In contrast, the items in an extension family represent queries to the extension oracle, and thus are pairs (T,ℓ) of a subset *T* of *U* and a non-negative integer ℓ. A reduction to a construction from [3] is used to build the extension family, and the extension family itself can be trivially used to attain a β-approximation algorithm for $\text {WeightedSM}$.

### Definition 5.1

*(Extension Family)* Let *U* be a finite set and $\textbf{w}:U\rightarrow \mathbb {N}$ be a weight function. Furthermore, let $\alpha ,\beta \ge 1$. We say ${\mathcal {E}}\subseteq 2^U\times \mathbb {N}$ is an $(\alpha ,\beta )$*-extension family* of *U* and $\textbf{w}$ if for every S⊆U there exists $(T,\ell ) \in {\mathcal {E}}$ which satisfies the following:

9 $$\begin{aligned} \begin{aligned} {\left| S\setminus T\right| }&\le \ell ,\\ \textbf{w}(T) + \alpha \cdot \textbf{w}(S\setminus T)&\le \beta \cdot \textbf{w}(S). \end{aligned} \end{aligned}$$

Let c≥1. The *c-cost* of an $(\alpha ,\beta )$-extension family ${\mathcal {E}}$ of *U* and $\textbf{w}$ is defined as $\text {\texttt {cost}}_c({\mathcal {E}}) :=\sum _{(T,\ell )\in {\mathcal {E}}} c^{\ell }$. The proof of Theorem 2.8 relies on the following lemma.

### Lemma 5.2

Let $\alpha ,c\ge 1$, β>1. Then there is an algorithm which given a finite set *U* and a weight function $\textbf{w}:U\rightarrow \mathbb {N}$ returns an $(\alpha ,\beta )$-extension family ${\mathcal {E}}$ of *U* and $\textbf{w}$ such that $\text {\texttt {cost}}_c({\mathcal {E}}) = \text {\texttt {amls}}(\alpha ,c,\beta )^{n + o(n)}$. Furthermore, the running time of the algorithm is $\text {\texttt {amls}}(\alpha ,c,\beta )^{n + o(n)}$.

Before heading to the proof of Lemma 5.2, we show how the lemma can be used to prove Theorem 2.8.

### Proof of Theorem 2.8

Let $\alpha , c \ge 1$, β>1. Consider the following algorithm $\mathscr {A}$:

- Given the input $(U, \textbf{w}, \mathcal {F})$, use Lemma 5.2 with $\alpha , \beta$ and *c* to construct an $(\alpha , \beta )$-extension family ${\mathcal {E}}$ of *U* and $\textbf{w}$.
- Let $\text {\texttt {Ext}}$ be the α-extension oracle. For each $(T_i, \ell _i) \in {\mathcal {E}}$, use $\text {\texttt {Ext}}$ to compute $X_i :=\text {\texttt {Ext}}(T_i, \ell _i)$.
- Define $\mathcal {T} :=\{T_i \cup X_i \mid (T_i, \ell _i) \in {\mathcal {E}}\}$ and return a set in $\mathcal {T}$ with the minimum weight, i.e., a set $T \in \mathcal {T}$ such that $\textbf{w}(T) = \min \{\textbf{w}(Y) \mid Y \in \mathcal {T}\}$.

The algorithm $\mathscr {A}$ first creates an $(\alpha , \beta )$-extension family ${\mathcal {E}}$. Then it goes over all elements $(T_i, \ell _i) \in {\mathcal {E}}$ and queries the oracle with $(T_i, \ell _i)$. The running time of this algorithm in the (α,c)-extension model is therefore equal to the running time of the algorithm from Lemma 5.2 plus $c^{\ell _i}$ for every query $(T_i, \ell _i)$, i.e., the cost of the extension family ${\mathcal {E}}$. By Lemma 5.2 this value is at most

$$\begin{aligned} \text {\texttt {amls}}(\alpha ,c,\beta )^{n + o(n)} + \text {\texttt {cost}}_c({\mathcal {E}}) = \text {\texttt {amls}}(\alpha ,c,\beta )^{n + o(n)}. \end{aligned}$$

### Claim 5.3

The algorithm $\mathscr {A}$ is a deterministic β-approximation for $\text {WeightedSM}$ in the (α,c)-extension model.

### Proof

Let $R \in \mathcal {T}$ be the set returned by the algorithm. Note that by definition of $\mathcal {T}$, $R = T_j \cup X_j$ for some $(T_j, \ell _j) \in {\mathcal {E}}$ and $X_j = \text {\texttt {Ext}}(T_j, \ell _j)$. In particular, $R = T_j \cup X_j = T_j \cup \text {\texttt {Ext}}(T_j, \ell _j) \in \mathcal {F}$ (see Definition 2.4). Let $S \in \mathcal {F}$ be a set with minimum weight in $\mathcal {F}$, i.e., *S* is a set in $\mathcal {F}$ which satisfies

$$\begin{aligned} \textbf{w}(S) = \min \{\textbf{w}(Y) \mid Y \in \mathcal {F}\} = \texttt {opt}(U,\textbf{w},{\mathcal {F}}). \end{aligned}$$

Since ${\mathcal {E}}$ is an $(\alpha , \beta )$-extension family, there exists $(T_i, \ell _i) \in {\mathcal {E}}$ such that $S, T_i$ and $\ell _i$ satisfy (9) for $T = T_i$ and $\ell = \ell _i$. Moreover, observe that

$$\begin{aligned} S \setminus T_i \in \{X \subseteq U \mid {\left| X\right| } \le \ell _i, X \cup T_i \in \mathcal {F}\} \end{aligned}$$

because $S \subseteq \left( S \setminus T_i \right) \cup T_i \in \mathcal {F}$. Hence by the definition of $\text {\texttt {Ext}}$ and $(T_i, \ell _i)$ it follows that

$$\begin{aligned} \textbf{w}(X_i) = \textbf{w}\left( \text {\texttt {Ext}}(T_i,\ell _i)\right)&\le \alpha \cdot \min \left\{ \textbf{w}(X)~|~X\subseteq U,~{\left| X\right| }\le \ell _i,~X\cup T_i\in {\mathcal {F}}\right\} \\&\le \alpha \cdot \textbf{w}\left( S \setminus T_i \right) \\&\le \beta \cdot \textbf{w}(S) - \textbf{w}(T_i). \end{aligned}$$

Finally, since $R \in \mathcal {T}$ is the set returned by the algorithm, we have

$$\begin{aligned} \textbf{w}(R)&= \min \{\textbf{w}(Y) \mid Y \in \mathcal {T}\}\\&\le \textbf{w}(T_i \cup X_i) \\&\le \textbf{w}(T_i) + \textbf{w}(X_i) \\&\le \beta \cdot \textbf{w}(S)\\&= \beta \cdot \texttt {opt}(U,\textbf{w},{\mathcal {F}}) \end{aligned}$$

which proves the claim. □

This shows that the algorithm $\mathscr {A}$ is a deterministic β-approximation for $\text {WeightedSM}$ in the (α,c)-extension model with the running time given in Theorem 2.8. □

The proof of Lemma 5.2 relies on the following construction for extension families in the unweighted case which is implicitly given in [3].

### Lemma 5.4

( [3]) Let $\alpha ,c\ge 1$ and β>1. Then there is a deterministic algorithm which given a finite set *U*, returns an $(\alpha ,\beta )$-extension family ${\mathcal {E}}$ of *U* and the uniform weight function $\textbf{w}:U\rightarrow \{1\}$ such that $\text {\texttt {cost}}_c({\mathcal {E}}) \le \Bigl ( \text {\texttt {amls}}(\alpha ,c,\beta )\Bigr )^{n + o(n)}$. Furthermore, the running time of the algorithm is $\Bigl ( \text {\texttt {amls}}(\alpha ,c,\beta )\Bigr )^{n + o(n)}$.

In a manner analogous to Sect. 4, we begin by introducing Lemma 5.5, which presents a slightly weaker form of Lemma 5.2. Within Lemma 5.5, ζ takes the place of the previously mentioned β from Lemma 5.2. Subsequently, in the proof of Lemma 5.2, we set the value of ζ as a function of β.

### Lemma 5.5

Let *U* be a given finite set with $n :={\left| U\right| }$, $\textbf{w}:U\rightarrow \mathbb {N}$ be a weight function, $\alpha ,c\ge 1$, ζ>1 and $\delta :\mathbb {N}\rightarrow (0,1]$ be a function such that $\delta (n) = \Omega \left( 1 / \log (n)\right)$. Then, there exists a deterministic algorithm $\mathscr {A}$ that constructs an $\bigl (\alpha ,(1 + \delta (n)) \cdot \zeta \bigr )$-extension family ${\mathcal {E}}$ of *U* and $\textbf{w}$ such that $\text {\texttt {cost}}_c({\mathcal {E}}) \le \Bigl ( \text {\texttt {amls}}(\alpha ,c, \zeta )\Bigr )^{n + o(n)}$. Furthermore, the running time of the algorithm is $\Bigl ( \text {\texttt {amls}}(\alpha ,c,\zeta )\Bigr )^{n+o(n)}$.

The proof of Lemma 5.5 contains repeated arguments from the proof of Lemma 4.5. To enhance the overall readability and reduce the notational burden, we keep the common arguments in both proofs.

### Proof of Lemma 5.5

In the proof, γ,d (which are defined in Algorithm 1) and δ are functions of *n*, but we omit the dependence on *n* for the sake of readability. We claim that Algorithm 1 satisfies the conditions stated in the lemma.

**Algorithm 1:**
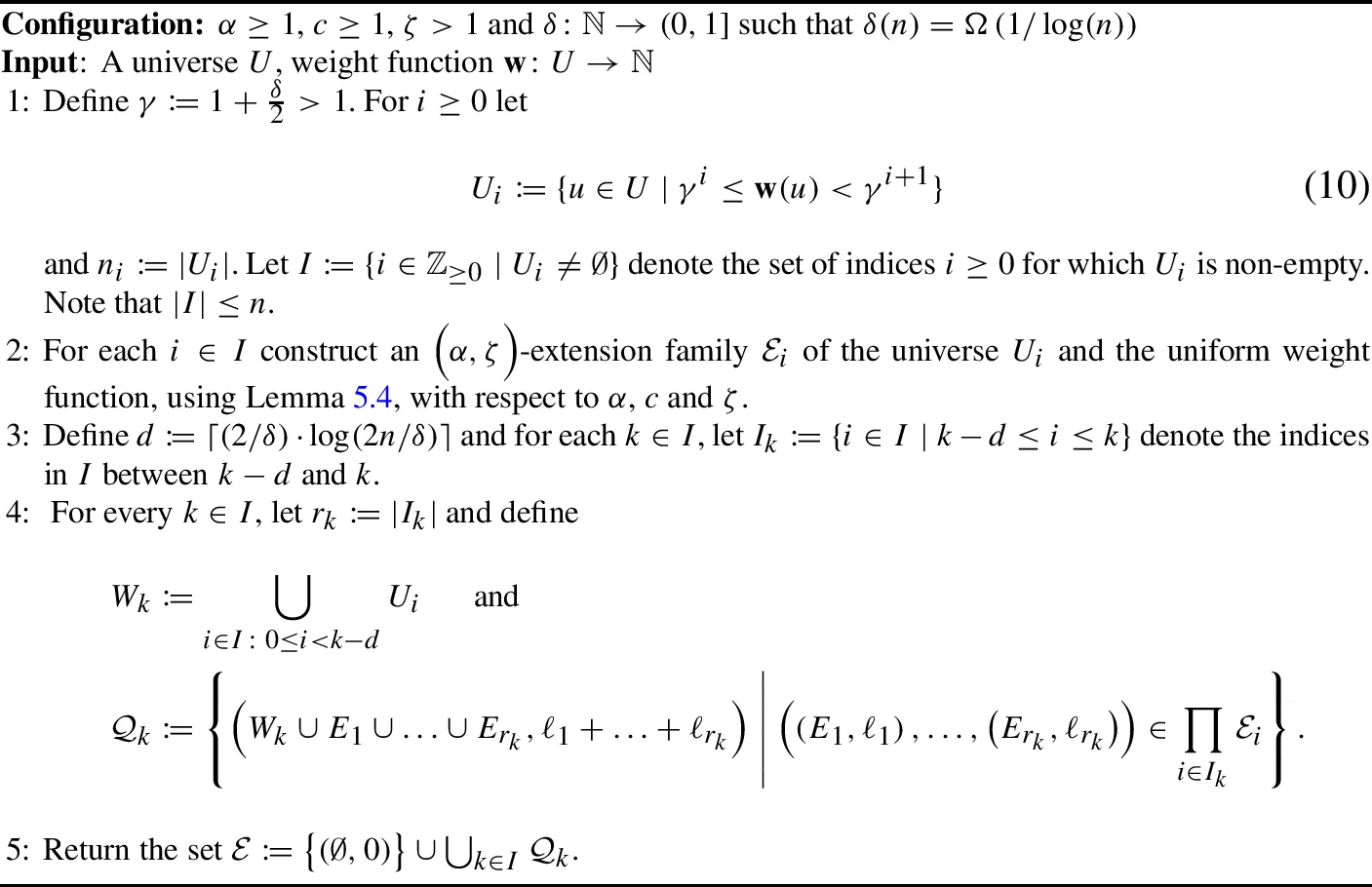
Extension Family for Arbitrary Weight Functions

### Claim 5.6

The set ${\mathcal {E}}$ returned by Algorithm 1 is an $\bigl ( \alpha , (1 + \delta ) \cdot \zeta \bigr )$-extension family of *U* and $\textbf{w}$.

### Proof

Let S⊆U be a set. We argue that there exists $(T,\ell ) \in {\mathcal {E}}$ such that *S*, *T* and ℓ satisfy (9). If S=∅, then we choose $(T,\ell ) = (\emptyset , 0) \in {\mathcal {E}}$, which satisfies (9). For the remainder, assume $S \ne \emptyset$. By the definition of γ and *d*, it holds that

11 $$\begin{aligned} \gamma ^d \ge \left( 1 + \frac{\delta }{2}\right) ^{(2/\delta ) \cdot \log (2n/\delta )} \ge 2^{\log (2n/\delta )} = \frac{2n}{\delta } \end{aligned}$$

using that $(1 + \frac{1}{x})^x \ge 2$ for all x≥1. Let k∈I be the largest index such that $S \cap U_k \ne \emptyset$. Recall that ${\left| W_k\right| } \le n$ and each $u \in W_k$ belongs to a set $U_i$ where i≤k-d-1. Hence, by the definition of $U_i$ in (10), we have

$$\begin{aligned} \textbf{w}(u) < \gamma ^{k - d - 1 + 1} = \gamma ^{k - d}, \end{aligned}$$

which implies that

$$\begin{aligned} \textbf{w}(W_k) < n\cdot \gamma ^{k - d}. \end{aligned}$$

Combining this with (11) and the choice of *k*, we obtain

12 $$\begin{aligned} \textbf{w}(W_k) \,<\, n \cdot \gamma ^{k - d} \,\le \, \frac{\delta }{2} \cdot \gamma ^{k} \,\le \, \frac{\delta }{2} \cdot \textbf{w}(S). \end{aligned}$$

The second inequality follows from (11) and finally the last inequality holds because by the definition of *k*, there exists $u \in S \cap U_k$ such that $\textbf{w}(S) \ge \textbf{w}(u) \ge \gamma ^{k}$ by (10).

For $i \in I_k$ let $S_i :=S \cap U_i$. Since ${\mathcal {E}}_i$ is an $(\alpha , \zeta )$-extension family of $U_i$ and the *uniform weight function*, for each $i \in I_k$ there exists $(T_i, \ell _i) \in {\mathcal {E}}_i$ such that

13 $$\begin{aligned} \begin{aligned} {\left| S_i \setminus T_i\right| }&\le \ell _i\\ {\left| T_i\right| } + \alpha \cdot {\left| S_i \setminus T_i\right| }&\le \zeta \cdot {\left| S_i\right| }. \end{aligned} \end{aligned}$$

Let $\overline{T} :=\bigcup _{i \in I_k} T_i$ and define

$$\begin{aligned} \begin{aligned} T&:=W_k \cup \overline{T}\\ \ell&:=\sum _{i \in I_k} \ell _i. \end{aligned} \end{aligned}$$

By definition of $\mathcal {Q}_k$ in Algorithm 1 it holds that $(T,\ell ) \in \mathcal {Q}_k \subseteq {\mathcal {E}}$. Observe that

14 $$\begin{aligned} \begin{aligned} S \setminus T&= \left( S \setminus T \right) \cap U\\&= \left( S \setminus T \right) \cap \left( W_k \cup \biggl ( \;\bigcup _{i \in I_k} U_i \biggr ) \right) \\&= \Bigl ( (S\setminus T) \cap W_k \Bigr ) \cup \left( (S \setminus T) \cap \biggl ( \;\bigcup _{i \in I_k} U_i \biggr ) \right) \\&= (S \setminus T) \cap \biggl ( \;\bigcup _{i \in I_k} U_i \biggr )\\&= \bigcup _{i \in I_k} \left( U_i \cap (S \setminus T)\right) \\&= \bigcup _{i \in I_k} S_i \setminus T_i \end{aligned} \end{aligned}$$

where the second equality follows from $S \subseteq \bigcup _{0 \le i \le k} U_i = W_k \cup \left( \bigcup _{k-d \le i \le k} U_i \right)$ and the fourth equality holds because $W_k \subseteq T$ which further implies $(S \setminus T) \cap W_k = \emptyset$. Therefore it holds that

$$\begin{aligned} {\left| S \setminus T\right| } = {\left| \bigcup _{i \in I_k} S_i \setminus T_i\right| } \le \sum _{i \in I_k} {\left| S_i \setminus T_i\right| } \le \sum _{i \in I_k} \ell _i = \ell . \end{aligned}$$

where the second inequality follows from (13).

For all $i \in I_k$ we also have that

15 $$\begin{aligned} \begin{aligned} \textbf{w}(T_i) + \alpha \cdot \textbf{w}(S_i \setminus T_i)&\le {\left| T_i\right| }\cdot \gamma ^{i+1} + \alpha \cdot \gamma ^{i+1} \cdot {\left| S_i \setminus T_i\right| }\\&\le \gamma ^{i+1} \cdot \left( {\left| T_i\right| } + \alpha \cdot {\left| S_i \setminus T_i\right| } \right) \\&\le \gamma ^{i+1} \cdot \zeta \cdot {\left| S_i\right| }\\&\le \zeta \cdot \gamma \cdot \textbf{w}(S_i) \end{aligned} \end{aligned}$$

where the third inequality follows from (13). Therefore we have that

$$\begin{aligned} \textbf{w}(T) + \alpha \cdot \textbf{w}(S\setminus T)&= \textbf{w}(W_k) + \textbf{w}\left( \bigcup _{i \in I_k} T_i \right) + \alpha \cdot \textbf{w}\left( S \setminus T \right) \\&\le \textbf{w}(W_k) + \sum _{i \in I_k} \Biggl ( \textbf{w}(T_i) + \alpha \cdot \textbf{w}(S_i \setminus T_i) \Biggr )  &   \text { by } (14)\\&< \frac{\delta }{2} \cdot \textbf{w}(S) + \sum _{i \in I_k} \Biggl ( \textbf{w}(T_i) + \alpha \cdot \textbf{w}(S_i \setminus T_i) \Biggr )  &   \text { by } (12) \\&\le \frac{\delta }{2} \cdot \textbf{w}(S) + \sum _{i \in I_k} \zeta \cdot \gamma \cdot \textbf{w}(S_i)  &   \text { by } (15) \\&< \zeta \cdot \frac{\delta }{2} \cdot \textbf{w}(S) + \sum _{i \in I_k} \zeta \cdot \gamma \cdot \textbf{w}(S_i)  &   \text { since } \zeta > 1 \\&\le \zeta \cdot \frac{\delta }{2} \cdot \textbf{w}(S) + \zeta \cdot \gamma \cdot \textbf{w}(S)\\&\le \left( 1 + \delta \right) \cdot \zeta \cdot \textbf{w}(S) \end{aligned}$$

which proves the claim. □

### Claim 5.7

$$\begin{aligned} \text {\texttt {cost}}_c({\mathcal {E}}) \le \Bigl ( \text {\texttt {amls}}(\alpha ,c,\zeta )\Bigr )^{n+o(n)}. \end{aligned}$$

### Proof

By Lemma 5.4, for each i∈I it holds that

16 $$\begin{aligned} \text {\texttt {cost}}_c({\mathcal {E}}_i) \le \Bigl ( \text {\texttt {amls}}(\alpha ,c,\zeta )\Bigr )^{n_i + o(n_i)}. \end{aligned}$$

For $i \in \mathbb {Z}_{\ge 0} \setminus I$ we define ${\mathcal {E}}_i = \{(\emptyset , 0)\}$. By doing so, we can make the assumption, without loss of generality, that ${\mathcal {E}}_i$ is nonempty for all $i \in \mathbb {Z}_{\ge 0}$. Note that this assumption does not have any effect on the value of $\text {\texttt {cost}}_c$ and it simplifies the following analysis. Also let $\textbf{E}$ denote the Cartesian product of ${\mathcal {E}}_{k-d}$ up to ${\mathcal {E}}_k$, i.e., $\textbf{E} :=\prod _{i \in \{k - d, \ldots , k\} } {\mathcal {E}}_i$.

With this assumption, for all k∈I we have that

17 $$\begin{aligned} \begin{aligned} \text {\texttt {cost}}_c(\mathcal {Q}_k)&= \sum _{\bigl ( (E_{k - d}, \ell _{k - d}), \ldots , (E_{k - 1}, \ell _{k - 1}), (E_{k}, \ell _{k}) \bigr ) \in \textbf{E}} c^{\ell _{k - d} + \ldots + \ell _{k - 1} + \ell _{k}}\\&= \sum _{\bigl ( (E_{k - d}, \ell _{k - d}), \ldots , (E_{k - 1}, \ell _{k - 1}), (E_{k}, \ell _{k}) \bigr ) \in \textbf{E}} c^{\ell _{k - d}} \cdot \ldots \cdot c^{\ell _{k - 1}} \cdot c^{\ell _k}\\&= \prod _{j = k -d}^{k} \;\sum _{(E,\ell ) \in {\mathcal {E}}_j}c^{\ell }\\&= \prod _{j = k - d}^{k} \;\text {\texttt {cost}}_c({\mathcal {E}}_j)\\&= \prod _{j \in I_k}\; \text {\texttt {cost}}_c({\mathcal {E}}_j) \end{aligned} \end{aligned}$$

Moreover, for every k∈I, we have

18 $$\begin{aligned} \begin{aligned} {\left| I_k\right| } \le d&\le c \cdot \frac{1}{\delta (n)} \cdot \log \left( \frac{n}{\delta (n)} \right) \\&\le c' \cdot \log (n) \cdot \log \left( n \cdot \log (n) \right) \\&= o(n) \end{aligned} \end{aligned}$$

where *c* and $c^{\prime }$ are constants, and the second inequality holds because $$\delta (n) = \Omega \left( \frac{1}{\log (n)} \right) $$.

By (16) and (17) we obtain

$$\begin{aligned} \text {\texttt {cost}}_c(\mathcal {Q}_k)&= \prod _{i \in I_k} \text {\texttt {cost}}_c\left( {\mathcal {E}}_i \right) \\&\le \prod _{i \in I_k} \Bigl ( \text {\texttt {amls}}(\alpha ,c,\zeta )\Bigr )^{n_i + o(n_i)}\\&= \Bigl ( \text {\texttt {amls}}(\alpha ,c,\zeta )\Bigr )^{n + o(n)}\\ \end{aligned}$$

where the last step follows from Lemma 4.4 and (18).

Finally, it holds that

$$\begin{aligned}&\text {\texttt {cost}}_c({\mathcal {E}}) \le \Biggl (1+\text {\texttt {cost}}_c \biggl (\;\bigcup _{k \in I} \mathcal {Q}_k \biggr )\Biggr ) \le \Biggl (1+\sum _{k \in I} \;\text {\texttt {cost}}_c\left( \mathcal {Q}_k \right) \Biggr ) \\&= \Bigl ( \text {\texttt {amls}}(\alpha ,c,\zeta )\Bigr )^{n + o(n)} \end{aligned}$$

since $${\left| I\right| } \le n$$. □

### Claim 5.8

The running time of Algorithm 1 is $\Bigl ( \text {\texttt {amls}}(\alpha ,c,\zeta )\Bigr )^{n+o(n)}$.

### Proof

The construction of $\{{\mathcal {E}}_i\}_{i \in I}$ takes time

$$\begin{aligned} \sum _{i \in I} \Bigl ( \text {\texttt {amls}}(\alpha ,c,\zeta )\Bigr )^{n_i + o(n_i)} \le \Bigl ( \text {\texttt {amls}}(\alpha ,c,\zeta )\Bigr )^{n+o(n)} \end{aligned}$$

by Lemma 5.4.

Finally, the construction of each $\mathcal {Q}_k$ takes time proportional to its size, which is upper bounded by $\text {\texttt {cost}}_c(\mathcal {Q}_k)$. Then, the construction of ${\mathcal {E}}$ takes at most time $\mathcal {O}(\text {\texttt {cost}}_c({\mathcal {E}}))$, where we have

$$\begin{aligned} \text {\texttt {cost}}_c({\mathcal {E}}) \le \Bigl ( \text {\texttt {amls}}(\alpha ,c,\zeta )\Bigr )^{n+o(n)} \end{aligned}$$

by Claim 5.7. All in all, the running time of Algorithm 1 is upper bounded by

$$\begin{aligned} \Bigl (\text {\texttt {amls}}(\alpha ,c,\zeta )\Bigr )^{n+o(n)}. \end{aligned}$$

The lemma follows from Claims 5.6 to 5.8.

Lastly, we use the following property of the function $\text {\texttt {amls}}$.□

### Lemma 5.9

For every fixed $\alpha \ge 1$ and c≥1, $\text {\texttt {amls}}(\alpha , c, x)$ is a continuous function of *x* on the interval (1,∞).

The proof of Lemma 5.9 is given Appendix C.

### Proof of Lemma 5.2

We claim that the algorithm $\mathcal {A}$ from Lemma 5.5 with $\zeta (n) = \beta - \frac{1}{\log (n)}$ and $\delta (n) = \frac{\beta }{\zeta (n)} - 1$ satisfies the properties listed in Lemma 5.2. Note that δ(n) and ζ(n) are functions of *n*, but in the following we write δ and ζ instead of δ(n) and ζ(n) for the sake of readability.

Observe that we have

$$\begin{aligned} \left( 1 + \delta \right) \cdot \zeta = \frac{\beta }{\zeta } \cdot \zeta = \beta . \end{aligned}$$

Hence, by Lemma 5.5, the set returned by $\mathcal {A}$ is an $(\alpha ,\beta )$-extension family ${\mathcal {E}}$ of *U* and $\textbf{w}$ such that $\text {\texttt {cost}}_c({\mathcal {E}}) = \text {\texttt {amls}}(\alpha ,c,\zeta )^{n + o(n)}$. Similarly, the running time of $\mathcal {A}$ is also bounded by $\text {\texttt {amls}}(\alpha ,c,\zeta )^{n + o(n)}$.

By Lemma 5.9, $\text {\texttt {amls}}(\alpha ,c,\zeta )$ is continuous in ζ. Therefore, by Lemma 4.9, it holds that $\text {\texttt {amls}}(\alpha ,c,\zeta )^{n + o(n)} = \text {\texttt {amls}}(\alpha ,c,\beta )^{n + o(n)}$ which proves the lemma. □

## Discussion

In this paper, we study weighted monotone subset minimization problems, where given a universe *U* with *n* elements and a weight function $\textbf{w}:U \rightarrow \mathbb {N}$, the goal is to find a subset S⊆U which satisfies a certain fixed property and has a minimum weight. For such problems, we show that the approximate monotone local search framework of Esmer et al. [3] can be extended to the weighted setting. In particular, given a parameterized α-approximate extension algorithm, that runs in time $c^k \cdot n^{\mathcal {O}(1)}$ and outputs a solution whose weight is at most $\alpha \cdot \textbf{w}(\texttt {OPT})$ where $\texttt {OPT}$ is a solution of size at most *k* and minimum weight, one can design an exponential β-approximation algorithm that runs faster than the proposed (natural) brute-force algorithm.

Note that for most of our applications, the parameterized approximation algorithms that are available in the literature [13–16, 19] provide bi-objective guarantees which are stronger than the requirements from the α-approximate extension algorithm. In particular, these algorithms run in time $c^k \cdot n^{\mathcal {O}(1)}$ and output a solution of size at most $\gamma \cdot k$ and weight at most $\alpha \cdot W$, if a solution of size at most *k* and weight at most *W* exists. That is, they (approximately) optimize the size and weight of the output solution *simultaneously*.

This leads to the following natural question. Consider more restrictive weighted monotone subset minimization problems where given a universe *U* on *n* vertices, a weight function $\textbf{w}$ on the elements of the universe, and a number *k*, the goal is to find a subset of the universe of size at most *k* that minimizes the weight and satisfies a certain fixed property. What is the analogue of brute-force in this setting? Can bi-objective parameterized approximation algorithms for weighted monotone subset minimization problems be used to design faster (than brute force) exponential approximation algorithms in this restrictive setting? What happens if we extend this setting to a bi-criteria optimization setting?

## Figures and Tables

**Fig. 3:**
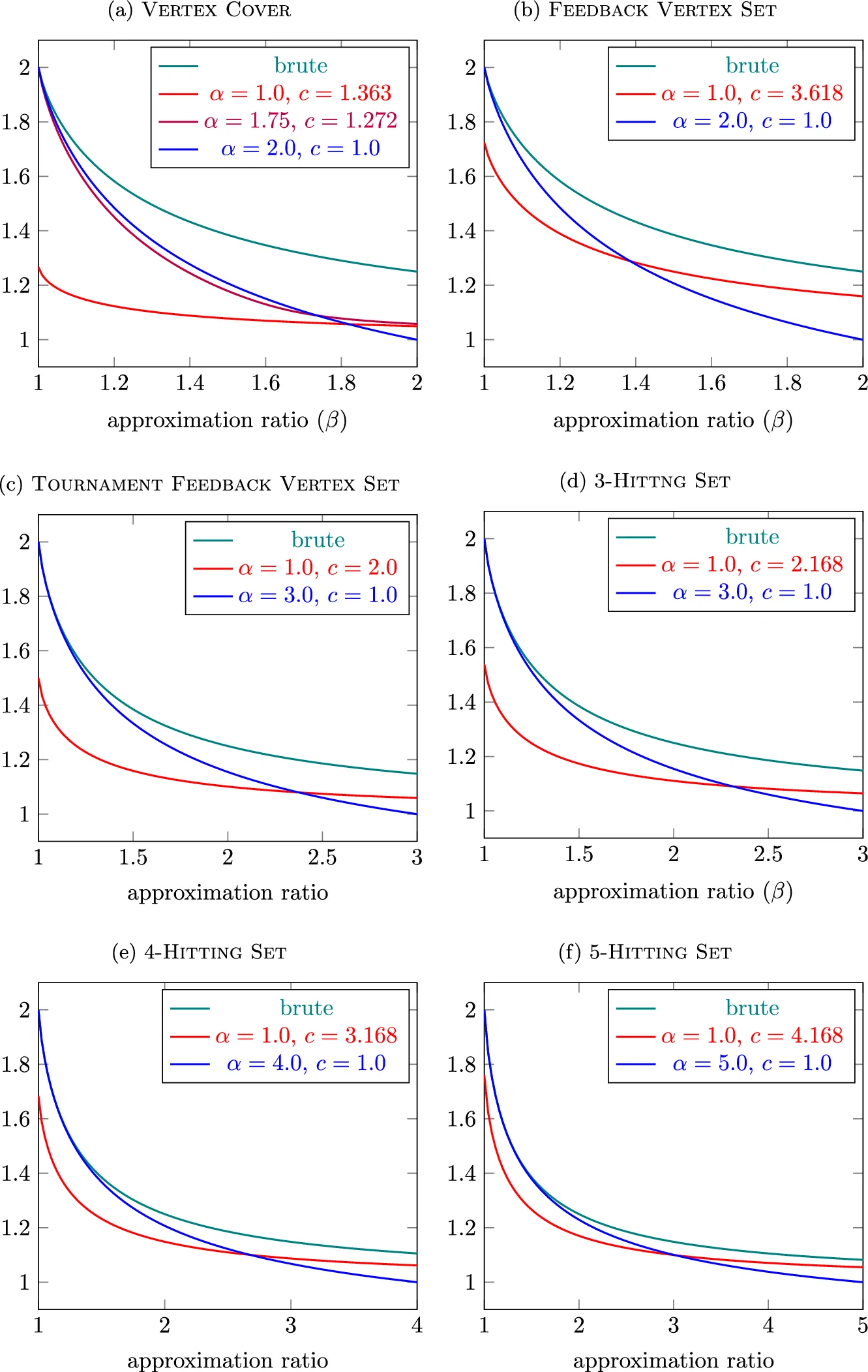
The figure shows running times for Vertex Cover, Feedback Vertex Set, Tournament Feedback Vertex Set, 3-Hitting Set, 4-Hitting Set and 5-Hitting Set. For each curve and approximation ratio β, the plotted value *d* denotes the base in the running time $d^{n + o(n)}$ of the corresponding β-approximation algorithm

**Fig. 4:**
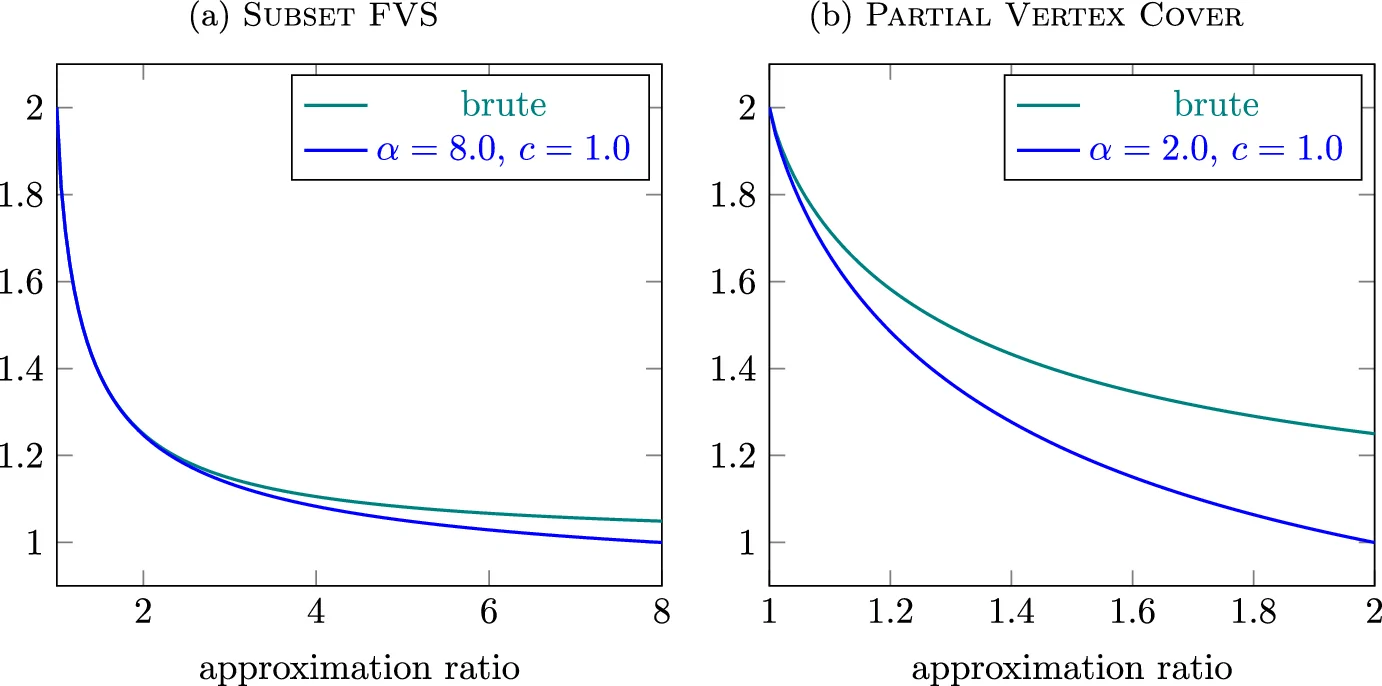
The figure shows running times for Subset FVS and Partial Vertex Cover. For each curve and approximation ratio β, the plotted value *d* denotes the base in the running time $d^{n + o(n)}$ of the corresponding β-approximation algorithm

## Data Availability

No datasets were generated or analysed during the current study.

## Appendix A Basics on O-Notation

In this section, we give a proof of Lemma 4.4.

### Lemma 4.4

Let $g,d:\mathbb {N}\rightarrow \mathbb {N}$ be two functions such that g∈n+o(n) and d∈o(n). We define $f:\mathbb {N}\rightarrow \mathbb {N}$ via

$$\begin{aligned} f(n) = \max _{n = n_1 + \dots + n_{d(n)}} \sum _{i=1}^{d(n)} g(n_i). \end{aligned}$$

Then f∈n+o(n).

### Proof

Let ε>0. Then there is some $N_g \in \mathbb {N}$ such that

$$\begin{aligned} g(n) \le \left( 1 + \frac{\varepsilon }{2}\right) \cdot n \end{aligned}$$

for every $n \ge N_g$. Let $C_g :=\max _{n < N_g} g(n)$ and define $\Gamma (n) = \{1 \le i \le d(n)\}$. Then

$$\begin{aligned} f(n)&= \max _{n = n_1 + \dots + n_{d(n)}} \sum _{i=1}^{d(n)} g(n_i)\\&= \max _{n = n_1 + \dots + n_{d(n)}} \sum _{i \in \Gamma (n):n_i \ge N_g} g(n_i) + \sum _{i \in \Gamma (n):n_i< N_g} g(n_i)\\&\le \max _{n = n_1 + \dots + n_{d(n)}} \sum _{i \in \Gamma (n):n_i \ge N_g} \left( 1 + \frac{\varepsilon }{2}\right) \cdot n_i + \sum _{i \in \Gamma (n) :n_i < N_g} C_g\\&\le \left( 1 + \frac{\varepsilon }{2}\right) \cdot n + d(n) \cdot C_g \end{aligned}$$

for every $n \in \mathbb {N}$. Since d∈o(n) we conclude that there is some $N_d$ such that

$$\begin{aligned} d(n) \le \frac{\varepsilon }{2 \cdot C_g} \cdot n \end{aligned}$$

for all $n \ge N_d$. So

$$\begin{aligned} f(n)&\le \left( 1 + \frac{\varepsilon }{2}\right) \cdot n + d(n) \cdot C_g\\&\le \left( 1 + \frac{\varepsilon }{2}\right) \cdot n + \frac{\varepsilon }{2 \cdot C_g} \cdot n \cdot C_g\\&= \left( 1 + \varepsilon \right) \cdot n \end{aligned}$$

for every $$n \ge N_d$$. □

## Appendix B The Definition of $\text {\texttt {amls}}$

The definition of $\text {\texttt {amls}}$ relies on several auxiliary functions. For every $\alpha ,\beta ,c\ge 1$ define  We follow the standard notation in which 0ln0=0 and ${\mathcal {H}}(0) = {\mathcal {H}}(1)=0$. We define $\text {\texttt {amls}}(\alpha ,c,\beta )$ by

$$\begin{aligned} \text {\texttt {amls}}(\alpha ,c,\beta ) :=\exp \left( \max _{~0\le \kappa \le \frac{1}{\beta } ~} ~\min _{~ M_{\alpha ,\beta }(\kappa ) \le \tau \le \beta \kappa ~}~ g_{\alpha ,\beta ,c}(\kappa ,\tau )\right) . \end{aligned}$$

Though $\text {\texttt {amls}}$ is defined using a max-min optimization problem, it is shown in [3] that $g_{\alpha ,\beta ,c}(\kappa ,\tau )$ is *convex* in τ for every fixed $0\le \kappa \le \frac{1}{\beta }$. Thus,

$$\begin{aligned} g_{\alpha ,\beta ,c}^*(\kappa ) = \min _{~M_{\alpha ,\beta }(\kappa ) \le \tau \le \beta \kappa ~}~ g_{\alpha ,\beta ,c}(\kappa ,\tau ) \end{aligned}$$

can be efficiently computed to arbitrary precision. It is also shown in [3] that $g_{\alpha ,\beta ,c}^*(\kappa )$ is *concave*. So its maximum can be efficiently computed to arbitrary precision as well. Together, the convexity and concavity properties allow for an efficient computation of $\text {\texttt {amls}}$.

## Appendix C Continuity properties

### Lemma 4.9

Let $f :I \rightarrow (1,\infty )$ be a continuous function on an open interval $I\subseteq \mathbb {R}$, $\alpha \in I$ with α>1 and let $g :\mathbb {N}\rightarrow \mathbb {N}$ such that g∈o(n). Define $\beta (n) :=\alpha - \frac{1}{\log (n)}$ for all $n \in \mathbb {N}$. Then it holds that

$$\begin{aligned} f\bigl (\beta (n)\bigr )^{n + g(n)} = f(\alpha )^{n + o(n)}. \end{aligned}$$

### Proof

Decompose $f\left( \beta (n) \right) ^{n + g(n)}$ into two terms: $f\left( \beta (n) \right) ^{n}$ and $f\left( \beta (n) \right) ^{g(n)}$. We have that

$$\begin{aligned} f\Bigl (\beta (n)\Bigr )^{n}&= f\left( \alpha \right) ^{n} \cdot \left( \frac{f\Bigl (\beta (n)\Bigr )}{f\left( \alpha \right) } \right) ^{n} \\&= f\left( \alpha \right) ^{n} \cdot 2^{E(n)} \end{aligned}$$

where $$E(n) :=n \cdot \log \left( \frac{f(\beta (n))}{f(\alpha ) }\right) $$.

### Claim C.1

$$\begin{aligned} E(n) \in o(n). \end{aligned}$$

### Proof

To prove the claim, let us calculate

$$\begin{aligned} \lim _{n \rightarrow \infty } \frac{E(n)}{n}= &   \lim _{n \rightarrow \infty } \log \left( \frac{f\Bigl (\beta (n)\Bigr )}{f\left( \alpha \right) } \right) = \log \left( \frac{\lim _{n \rightarrow \infty } f\Bigl (\beta (n)\Bigr )}{f(\alpha )} \right) = \log \left( \frac{f(\alpha )}{f(\alpha )} \right) \nonumber \\= &   0 \end{aligned}$$

where the second and second to last step follows from the continuity of the functions log and *f*, respectively. □

Similarly, we have

$$\begin{aligned} f\left( \beta (n) \right) ^{g(n)} = f\left( \alpha \right) ^{g(n)} \cdot 2^{F(n)} \end{aligned}$$

where $$F(n) :=g(n) \cdot \log \left( \frac{f(\beta (n))}{f\left( \alpha \right) } \right) $$.

### Claim C.2

$$\begin{aligned} F(n) \in o(n). \end{aligned}$$

### Proof

Similar to the Claim C.1, we calculate

19 $$\begin{aligned} \begin{aligned} \lim _{n \rightarrow \infty } \frac{F(n)}{n}&= \lim _{n \rightarrow \infty } \frac{g(n)}{n} \cdot \log \left( \frac{f\left( \beta (n) \right) }{f(\alpha )} \right) \\&= \biggl ( \lim _{n \rightarrow \infty } \frac{g(n)}{n} \biggr ) \cdot \biggl ( \lim _{n \rightarrow \infty } \log \left( \frac{f\left( \beta (n) \right) }{f(\alpha )} \right) \biggr )\\&= 0. \end{aligned} \end{aligned}$$

□

Combining everything, we get

$$\begin{aligned} f\left( \beta (n) \right) ^{n + g(n)}&= f(\alpha )^{n} \cdot 2^{E(n) + F(n)} \cdot f(\alpha )^{g(n)}\\&= f(\alpha )^{n + g(n) + \frac{E(n) + F(n)}{\log _2 f(\alpha )} }\\&= f\left( \alpha \right) ^{n + o(n)}. \end{aligned}$$

□

In the following, we state some definitions and claims given in [23] which we use in the proof of Lemma 5.9.

Let $T \subseteq \mathbb {R}^{p}$ be an open set. For each t∈T, let *P*(*t*) be the following optimization problem

$$\begin{aligned} P(t) :\min _{x \in \mathbb {R}^{n}} f(x,t) \text { s.t. } x \in F(t) :=\{ x \in \mathbb {R}^{n} \mid g_j(x,t) \le 0, j \in \{1, \ldots , m\} \}, \end{aligned}$$

for continuous functions *f* and $g_j$, for some m>0. Let $v(t) = \min _{x \in F(t)} f(x,t)$ denote the global minimum of *P*(*t*) and let *S*(*t*) be the set of global minimizers of *P*(*t*), i.e., $S(t) = \{x \in F(t) \mid f(x,t) = v(t)\}$.

In [23], two sufficients conditions are given to show that *v*(*t*) is upper semi-continuous and lower semi-continuous, respectively.

### Lemma C.3

([23]) Let t∈T. If there exists a $\delta _{t} > 0$ and a compact set $C_t$ such that

$$\begin{aligned} \bigcup _{\Vert {t' - t}\Vert < \delta _t} F(t') \subseteq C_{t}, \end{aligned}$$

then *v* is lower semi-continuous at *t*.

### Lemma C.4

( [23]) Let t∈T. If there exists x∈S(t) such that there is a sequence $\left( x_{\ell } \right) _{\ell \in \mathbb {N}} \rightarrow x$ which satisfies

$$\begin{aligned} g_j\left( x_\ell , t \right) < 0 \quad \forall j \in \{1, \ldots , m\}, \;\forall \ell \in \mathbb {N}, \end{aligned}$$

then *v* is upper semi-continuous at *t*.

Now we are ready to prove Lemma 5.9.

### Lemma 5.9

For every fixed $\alpha \ge 1$ and c≥1, $\text {\texttt {amls}}(\alpha , c, x)$ is a continuous function of *x* on the interval (1,∞).

### Proof

Define $T_1 = \{(\beta , \kappa ) \mid \beta > 1, 0< \kappa < 2\}$. Moreover, also define the functions $M_{\alpha }(\beta , \kappa ) :=M_{\alpha , \beta }(\kappa )$ and

$$\begin{aligned} h_1(\beta , \kappa )&= {\left\{ \begin{array}{ll} \frac{1}{\beta } &  \text { if } \kappa> \frac{1}{\beta }\\ \kappa & \text { otherwise} \end{array}\right. }\\ h_2(\beta , \kappa , \tau )&= {\left\{ \begin{array}{ll} M_{\alpha }(\beta , \kappa ) &  \text { if } \tau < M_\alpha (\beta , \kappa )\\ \beta \cdot \kappa &  \text { if } \tau > \beta \cdot \kappa \\ \tau & \text { otherwise}. \end{array}\right. } \end{aligned}$$

For each $(\beta , \kappa ) \in T_1$ write

$$\begin{aligned}  &   P_1(\beta , \kappa ) :\min _{\tau \in \mathbb {R}} \tilde{g}(\beta , \kappa , \tau ) \; \text { s.t. } \tau \in F_1(\beta , \kappa ) \nonumber \\    &   :=\{\tau \in \mathbb {R} \mid \tau - \beta \cdot \kappa \le 0 , -t + M_{\alpha }(\beta , \kappa ) \le 0\} \end{aligned}$$

where $\tilde{g}\left( \beta , \kappa , \tau \right) = g_{\alpha , \beta , c} \Bigl (h_1\left( \beta , \kappa \right) , h_2\left( \beta , \kappa , \tau \right) \Bigr )$. Recall that $M_{\alpha , \beta }(\kappa )$ and $g_{\alpha , \beta , c}(\kappa ,\tau )$ are both defined in Appendix B.

Note that $\tilde{g}$ is well defined for all $(\beta , \kappa ) \in T_1$ and $\tau \in \mathbb {R}$. Moreover, since the entropy function is continuous and $g_{\alpha , \beta , c}(\kappa ,\tau )$ consists of elementary arithmetic operations and composition of continuous functions, $g_{\alpha , \beta , c}(\kappa ,\tau )$ is a continuous function of β>1, $0< \kappa < \frac{1}{\beta }$ and $\tau \in [M_\alpha (\beta , \kappa ), \beta \cdot \kappa ]$. Finally, since $h_1$ and $h_2$ are continuous functions, their composition with *g*, i.e., the function $\tilde{g}$, is also continuous. Finally, the functions $\tau - \beta \cdot \kappa$ and $-\tau + M_{\alpha }(\beta , \kappa )$ are also continuous since they consist of elementary arithmetic operations.

Let $v_1$ be the function that maps $(\beta , \kappa ) \in T_1$ to the global minimum of $P_1(\beta , \kappa )$.

### Claim C.5

$v_1$ is lower semi-continuous at $(\beta ,\kappa )$ for all $(\beta , \kappa ) \in T_1$.

### Proof

Let $(\beta ,\kappa ) \in T_1$. Observe that for all $(\beta ',\kappa ') \in T_1$, it holds that $F_1(\beta ',\kappa ') \subseteq [0,1]$. Let δ be an arbitrarily small number. We have that

$$\begin{aligned} \bigcup _{\Vert {(\beta , \kappa ) - (\beta ', \kappa ')}\Vert < \delta } F_1(\beta ', \kappa ') \subseteq [0,1], \end{aligned}$$

therefore by Lemma C.3, $v_1$ is lower semi-continuous at $(\beta , \kappa )$. □

### Claim C.6

$v_1$ is upper semi-continuous at $(\beta ,\kappa )$ for all $(\beta , \kappa ) \in T_1$.

### Proof

Let $(\beta ,\kappa ) \in T_1$. Observe that any global minimizer τ of $P_1(\beta , \kappa )$ lies in the interval $[M_{\alpha }(\beta , \kappa ), \beta \cdot \kappa ]$. Suppose $\tau \ne M_{\alpha }(\beta , \kappa )$, in that case the sequence $x_\ell :=\tau - \frac{1}{\ell }$ converges to τ and satisfies $M_\alpha (\beta , \kappa )< x_\ell < \beta \cdot \kappa$ for large enough ℓ. If $\tau = M_{\alpha }(\beta , \kappa )$, then the same argument works for $x_\ell :=\tau + \frac{1}{\ell }$. Therefore by Lemma C.4 it holds that $v_1$ is upper semi-continuous at $(\beta ,\kappa )$. □

Claims C.6 and C.5 together imply that $v_1$ is continuous on $T_1$.

### Claim C.7

$v_1$ is also continuous at (β,0) for β>1.

### Proof

Let $\left( \beta _\ell , \kappa _\ell \right) _{\ell \in \mathbb {N}}$ be a sequence such that $\beta _\ell > 1$, $\kappa _\ell \ge 0$ and $\left( \beta _\ell , \kappa _\ell \right) \rightarrow \left( \beta , 0 \right)$. For each $\ell \in \mathbb {N}$, let $\tau ^{*}_{\ell }$ be the value such that $v_1(\beta _\ell , \kappa _\ell ) = \tilde{g}(\beta _{\ell }, \kappa _\ell , \tau ^{*}_{\ell })$. Note that since $M_\alpha (\beta _\ell , \kappa _\ell ) \le \tau ^{*}_{\ell } \le \beta _\ell \cdot \kappa _\ell$ and

$$\begin{aligned} \lim _{\ell \rightarrow \infty } M_\alpha (\beta _\ell , \kappa _\ell ) = 0 = \lim _{\ell \rightarrow \infty } \beta _\ell \cdot \kappa _\ell , \end{aligned}$$

it holds that $\lim _{\ell \rightarrow \infty } \tau ^{*}_{\ell } = 0$ by the sandwich theorem. Therefore, by using the continuity of the function $\tilde{g}$, we find that

$$\begin{aligned} \lim _{\ell \rightarrow \infty } v_1(\beta _\ell , \kappa _\ell ) = \lim _{\ell \rightarrow \infty } \tilde{g}(\beta _\ell , \kappa _\ell , \tau ^{*}_{\ell } ) = \tilde{g}(\beta , 0, 0) = v_1(\beta , 0), \end{aligned}$$

where the last equality holds because $F_1(\beta , 0) = \{0\}$. □

In particular, we get that $v_1$ is continuous on the set $\{(\beta , \kappa ) \mid \beta > 1, 0 \le \kappa \le \frac{1}{\beta }\}$.

In a similar manner, let us now define $T_2 :=\{\beta \in \mathbb {R} \mid \beta > 1\}$ and for each $\beta \in T_2$ write

$$\begin{aligned} P_2(\beta ) :\max _{\kappa \in \mathbb {R}}\; v_1(\beta , \kappa ) \text { s.t. } \kappa \in F_2(\beta ) :=\left\{ \kappa \in \mathbb {R} \mid -\kappa \le 0,\; \kappa - \frac{1}{\beta } \le 0\right\} . \end{aligned}$$

Recall that the function that is maximized, that is $v_1$, is already shown to be continuous. Moreover, it can be verified that the constraint functions are also continuous.

Let $v_2$ be the function that maps $\beta \in T_2$ to the global maximum of $P_2(\beta )$. Note that for $\alpha \ge 1$ and c≥1, by the definition of $\text {\texttt {amls}}$ it holds that $v_2(\beta ) = \text {\texttt {amls}}(\alpha , c, \beta )$.

### Claim C.8

$v_2$ is lower semi-continuous at β for all $\beta \in T_2$.

### Proof

Let $\beta \in T_2$. Observe that for all $\beta ' \in T_2$ it holds that $F_2(\beta ') \subseteq [0,1]$. Let δ be an arbitrarily small number. We have that

$$\begin{aligned} \bigcup _{\Vert {\beta - \beta '}\Vert < \delta } F_2(\beta ') \subseteq [0,1], \end{aligned}$$

therefore by Lemma C.3, $v_2$ is lower semi-continuous at β. □

### Claim C.9

$v_2$ is upper semi-continuous at β for all $\beta \in T_2$.

### Proof

Let $(\beta ,\kappa ) \in T_1$. Observe that any global minimizer κ of $P_2(\beta )$ lies in the interval $[0, \frac{1}{\beta }]$. Suppose κ>0, in that case the sequence $x_\ell :=\kappa - \frac{1}{\ell }$ converges to κ and satisfies $0< x_\ell < \kappa \le \frac{1}{\beta }$ for large enough ℓ. If κ=0, then the same argument works for $x_\ell :=\kappa + \frac{1}{\ell }$. Therefore by Lemma C.4 it holds that $v_2$ is upper semi-continuous at κ.

□

Claims C.8 and C.9 together imply that $v_2$ is continuous on $T_2$, which proves the lemma. □

## Appendix D Problem Definitions

In this section, we give the problem definitions of all the problems discussed in the paper. For simplicity, we define the problems in their unweighted version. In the weighted version, the vertices are equipped with weights and we are looking for a solution *S* of minimum weight.

For the next problems, we require some additional definitions. A graph *G* is *cluster graph* if every connected component of *G* is a complete graph. A *cograph* is a graph *G* which does not contain $P_4$ (a path on 4 vertices) is an induced subgraph. Finally, a graph *G* is a *split graph* if the vertex set can be partitioned into two sets V(G)=I⊎C such that *I* is an independent set and *C* is a clique in *G*.

## Running Times

We provide extensive data sets on the running times for the obtained exponential approximation algorithms for the problems listed in Sect. 3. Table 3 contains the running times for selected approximation ratios, and graphical visualizations can be found in Figures 3 and 4.

**Table 3:** An entry *d* in column β means that the respective algorithm outputs a β-approximation in time $d^{n + o(n)}$.

Vertex Cover									
	1.1	1.2	1.3	1.4	1.5	1.6	1.7	1.8	1.9
$\text {\texttt {brute}}$	1.716	1.583	1.496	1.433	1.385	1.347	1.317	1.291	1.269
(α=1,c=1.363)	1.158	1.123	1.103	1.089	1.078	1.07	1.064	1.058	1.054
(α=2,c=1)	1.659	1.485	1.366	1.277	1.208	1.151	1.104	1.064	1.03

Feedback Vertex Set									
	1.1	1.2	1.3	1.4	1.5	1.6	1.7	1.8	1.9
$\text {\texttt {brute}}$	1.716	1.583	1.496	1.433	1.385	1.347	1.317	1.291	1.269
(α=1,c=3.618)	1.489	1.39	1.327	1.283	1.25	1.225	1.204	1.187	1.172
(α=2,c=1)	1.659	1.485	1.366	1.277	1.208	1.151	1.104	1.064	1.03

Tournament Feedback Vertex Set									
	1.2	1.4	1.6	1.8	2.0	2.2	2.4	2.6	2.8
$\text {\texttt {brute}}$	1.583	1.433	1.347	1.291	1.25	1.22	1.196	1.177	1.162
(α=1,c=2)	1.251	1.181	1.143	1.119	1.102	1.089	1.079	1.071	1.065
(α=3,c=1)	1.566	1.393	1.286	1.211	1.155	1.111	1.076	1.047	1.022

3-Hitting Set									
	1.2	1.4	1.6	1.8	2.0	2.2	2.4	2.6	2.8
$\text {\texttt {brute}}$	1.583	1.433	1.347	1.291	1.25	1.22	1.196	1.177	1.162
(α=1,c=2.168)	1.274	1.197	1.156	1.13	1.111	1.097	1.086	1.078	1.071
(α=3,c=1)	1.566	1.393	1.286	1.211	1.155	1.111	1.076	1.047	1.022

4-Hitting Set									
	1.3	1.6	1.9	2.2	2.5	2.8	3.1	3.4	3.7
$\text {\texttt {brute}}$	1.496	1.347	1.269	1.22	1.186	1.162	1.143	1.128	1.116
(α=1,c=3.168)	1.305	1.209	1.16	1.13	1.11	1.095	1.084	1.075	1.068
(α=4,c=1)	1.489	1.325	1.231	1.168	1.122	1.087	1.059	1.036	1.017

5-Hitting Set									
	1.4	1.8	2.2	2.6	3.0	3.4	3.8	4.2	4.6
$\text {\texttt {brute}}$	1.433	1.291	1.22	1.177	1.149	1.128	1.112	1.1	1.09
(α=1,c=4.168)	1.302	1.199	1.149	1.12	1.1	1.086	1.076	1.067	1.061
(α=5,c=1)	1.43	1.276	1.193	1.139	1.101	1.072	1.048	1.03	1.014

Subset FVS									
	1.7	2.4	3.1	3.8	4.5	5.2	5.9	6.6	7.3
$\text {\texttt {brute}}$	1.317	1.196	1.143	1.112	1.093	1.079	1.069	1.061	1.055
(α=8,c=1)	1.316	1.19	1.129	1.092	1.066	1.046	1.031	1.024	1.009

Partial Vertex Cover									
	1.1	1.2	1.3	1.4	1.5	1.6	1.7	1.8	1.9
$\text {\texttt {brute}}$	1.716	1.583	1.496	1.433	1.385	1.347	1.317	1.291	1.269
(α=2,c=1)	1.659	1.485	1.366	1.277	1.208	1.151	1.104	1.07	1.03

## Footnotes

1 The $\mathcal {O}^*$ notation hides polynomial factors in *n*.

2 An earlier version of [4, Theorem 5.1] claimed a stronger bound. The authors’ revised version gives a corrected, slightly weaker statement, which is used here.
